# Patterns of Soil Microbial Diversity, Assembly, and Co-Occurrence Along a Natural Salinity Gradient in an Inland Saline–Alkali Wetland

**DOI:** 10.3390/microorganisms14071602

**Published:** 2026-07-22

**Authors:** Jie Wei, Fan Chang, Haomin Yang, Yan Sun, Zhi Li, Jun Li, Nannan Liu, Zhuan Hao

**Affiliations:** 1 College of Environmental and Life Sciences, Weinan Normal University, Weinan 714009, China; lijun@wnu.edu.cn (J.L.); haozhuan1987@163.com (Z.H.); 2 Shaanxi Institute of Microbiology, Xi’an 710043, China; fox387@163.com; 3 Fisheries Institute, Sichuan Academy of Agricultural Sciences, Yibin 644000, China; yhm17808232385@163.com; 4 College of Life Sciences, Shaanxi Normal University, Xi’an 710119, China; sunyan@snnu.edu.cn (Y.S.); lizhi@snnu.edu.cn (Z.L.); 5 Key Laboratory for Ecology and Environment of River Wetlands in Shaanxi Province, Weinan 714009, China; liunan2008.hi@163.com

**Keywords:** saline–alkali wetland, microbial community assembly, deterministic selection, co-occurrence network

## Abstract

Natural inland saline–alkaline wetlands offer opportunities for evaluating microbial responses to long-term salinity stress. This study examined surface soils from non-saline, moderately saline, and hypersaline sites in the Luyang Lake wetland, measuring comprehensive edaphic variables (including SAR, ESP, carbonate/bicarbonate chemistry, moisture, DOC, and inorganic N) alongside bacterial and fungal communities via 16S rRNA and ITS sequencing. A coupled salinity–ion and nutrient gradient was identified, with hypersaline soils characterized by high Na^+^, Cl^−^, SAR, and ESP alongside depleted organic carbon and nitrogen. Bacterial α-diversity exhibited a significant unimodal response along the salinity gradient (quadratic regression: *p* < 0.001), peaking at moderate salinity. Fungal Shannon diversity declined with increasing salinity, but fungal Chao1 richness showed a U-shaped response, highlighting domain-specific and metric-dependent patterns along the gradient. Community assembly analyses revealed contrasting dynamics: deterministic processes were more prevalent in bacterial assembly in hypersaline soils, while fungal assembly remained predominantly stochastic. Co-occurrence networks showed sparser topological structure in high-salinity soils. These patterns are consistent with domain-specific microbial variation along the gradient to coupled edaphic stressors in inland saline–alkaline wetlands.

## 1. Introduction

Saline–alkali wetlands are important transitional ecosystems between terrestrial and aquatic environments. Inland saline–alkali wetlands differ substantially from coastal salt marshes and secondary salinized agricultural soils in both formation processes and environmental constraints. Coastal saline wetlands are largely influenced by seawater intrusion and tidal exchange, whereas secondary salinization in farmland is often associated with irrigation, fertilization, and tillage. In contrast, naturally developed inland saline–alkali wetlands commonly occur in closed or semi-closed basins, where strong evaporation, shallow groundwater movement, poor drainage, and long-term ion accumulation jointly promote primary salinization and alkalization. As a result, soil salinity in these systems is often coupled with elevated alkalinity, ionic imbalance, nutrient depletion, sparse vegetation cover, and hydrological isolation [1]. These features are associated with altered soil structure, nutrient availability, vegetation development, and belowground biodiversity [2]. In inland saline–alkaline wetlands, the ecological roles of soil microorganisms are shaped not only by the direct osmotic and ionic stress of elevated salinity and sodicity, but also by the concomitant depletion of available carbon and nutrients, as well as by shifts in halophytic vegetation that alter rhizosphere inputs and soil redox conditions [3]. Although many studies have examined microbial responses to salinity in agricultural soils, less is known about microbial diversity, community assembly, and association patterns in naturally developed inland saline–alkali wetlands [4]. Examining microbial communities along natural salinity gradients can advance understanding of how long-term salinization is associated with belowground ecological processes.

Soil microbiomes are sensitive to changes in physicochemical conditions [5]. Soil pH, salinity, nutrient availability, moisture, and texture can influence microbial survival, reproduction, dispersal, and niche differentiation, thereby shaping community composition and diversity [6]. Beyond describing taxonomic variation, identifying community assembly processes is important for understanding how environmental filtering and ecological drift jointly regulate microbial community structure. Ecological theory distinguishes deterministic processes, including homogeneous and heterogeneous selection, from stochastic processes, such as dispersal limitation, homogenizing dispersal, and undominated processes [7,8]. Quantifying the relative importance of these processes can help determine whether observed community differences are more strongly associated with niche-based selection or with neutral processes such as random colonization, extinction, and dispersal [9]. In saline soils with strong gradients in salinity, alkalinity, and resource availability, deterministic selection may become more prominent relative to stochastic processes, though this prediction has not been extensively tested in inland wetland systems [10]. Conversely, in less stressful or more environmentally homogeneous habitats, stochastic processes may contribute more substantially to community turnover [11]. The balance between deterministic and stochastic processes may differ between bacterial and fungal communities, but empirical evidence for this contrast in saline wetlands remains limited.

Microbial co-occurrence networks provide a framework for evaluating statistical association patterns among microbial taxa. Network edges represent significant correlations in abundance patterns and should be interpreted as statistical associations rather than direct evidence of ecological interactions [12]. Environmental stressors can influence microbial activity and shared environmental preferences, thereby modifying the structure of correlation-based networks [13]. High salinity is associated with reduced microbial richness, which may lead to sparser correlation networks, whereas moderate nutrient availability may support more complex statistical associations by increasing resource heterogeneity [14]. Network topological properties, such as node degree, modularity, and clustering coefficient, describe the structure of inferred association patterns [15]. Linking these properties with soil physicochemical variables may clarify how environmental gradients are associated with changes in microbial statistical associations [16]. An integrated analysis of microbial diversity, community assembly, and co-occurrence networks is needed to understand microbial responses to salinization in inland saline–alkali wetlands.

Luyang Lake is an inland wetland located in the eastern Guanzhong Plain of Shaanxi Province, China. The region lies in a climatic transition zone between arid/semi-arid and temperate monsoon conditions, where evaporation often exceeds precipitation. Unlike coastal wetlands affected by seawater intrusion or agricultural areas influenced by secondary salinization, salinity accumulation in Luyang Lake is mainly associated with natural evaporation, closed topography, groundwater movement, and ancient salt-lake deposits. The soils contain high background levels of soluble salts, including Na^+^, Cl^−^, and SO_4_^2−^, and the low-lying landscape favors surface salt accumulation. These conditions have produced a natural salinity gradient ranging from non-saline to moderately saline and hypersaline soils [17]. In this study, surface soils were collected from non-saline, moderately saline, and hypersaline zones of the Luyang Lake wetland. Soil physicochemical properties spanning salinity indicators (EC, SAR, ESP), pH, nutrient availability (AN, AP, AK), and ionic composition were quantified to characterize the edaphic stress gradient. Bacterial and fungal community structure were profiled via Illumina amplicon sequencing targeting the 16S rRNA V3-V4 region and ITS1 region, respectively. In saline–alkaline soils, microbial ecological functions are strongly constrained by ionic toxicity (particularly Na^+^ and Cl^−^), osmotic stress, and pH-induced nutrient limitation, which collectively filter community composition toward halotolerant and alkaliphilic taxa. Specifically, this study aimed to: (1) characterize changes in bacterial and fungal diversity and community composition along the natural salinity gradient; (2) identify the key soil physicochemical variables associated with microbial community variation; and (3) evaluate how microbial community assembly processes and co-occurrence network patterns vary among salinity zones. Due to higher physiological sensitivity of fungal hyphae to osmotic stress and stronger dependence on plant-derived carbon, we predict fungal diversity will decline more steeply than bacterial diversity along the salinity gradient. We also examined whether bacterial and fungal communities showed contrasting responses to the gradient, but we did not formulate a directional prediction for this comparison due to insufficient prior evidence. This study examines the ecological responses of bacterial and fungal communities to natural salinization in an inland saline–alkali wetland. We emphasize that this study employs a cross-sectional survey design along a natural gradient. Because salinity, vegetation type, and nutrient availability are inherently correlated in this system, their independent effects on microbial communities cannot be disentangled without manipulative experiments. The results should therefore be interpreted as descriptive patterns that generate testable hypotheses rather than as evidence of causal mechanisms.

## 2. Materials and Methods

### 2.1. Study Area and Sample Collection

The study was conducted in the Luyang Lake wetland, located in Pucheng County, Shaanxi Province, China (109°07′–109°51′ E, 34°41′–35°01′ N). This region has a semi-arid climate characterized by abundant sunshine, low and seasonally concentrated precipitation, and strong evaporation. Geomorphologically, the wetland occupies a low-lying depression with relatively poor drainage, where surface runoff and shallow groundwater can promote salt accumulation in surface soils.

**Figure 1:**
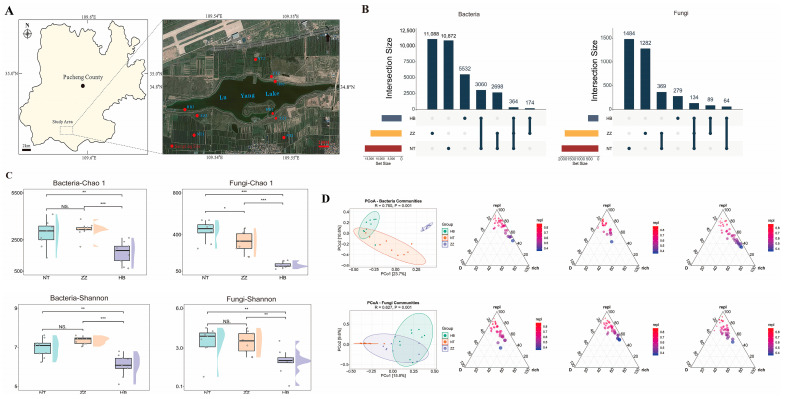
Study area and spatial patterns of microbial community diversity in the Luyang Lake wetland. (**A**) Map showing the geographical location of sampling sites across the salinity gradient: low salinity (NT), moderate salinity (ZZ), and high salinity (HB). (**B**) UpSet plots showing unique and shared bacterial (left) and fungal (right) ASVs across the three salinity groups. Horizontal bars indicate set sizes, vertical bars indicate intersection sizes, and the dot matrix denotes group membership. (**C**) Boxplots show raw quadrat values (*n* = 27); overlaid red diamonds indicate site means (*n* = 3 sites per group). Significance symbols denote LMM emmeans comparisons with Site as a random factor (Satterthwaite df ≈ 9), supplemented by Wilcoxon rank-sum tests where normality assumptions were violated. Quadratic regression on site-level means (*n* = 9) confirmed a significant unimodal relationship between bacterial α-diversity and salinity (Shannon: R^2^ = 0.58, *p* < 0.001; Chao1: R^2^ = 0.48, *p* < 0.001), with optima at intermediate EC. Fungal Shannon showed a weak quadratic fit consistent with a monotonic decline (R^2^ = 0.28, *p* = 0.02), whereas fungal Chao1 exhibited a U-shaped response (R^2^ = 0.71, *p* < 0.001; see Figure S1 for regression plots). Significance levels are indicated as * *p* < 0.05, ** *p* < 0.01, *** *p* < 0.001. ns, not significant (*p* ≥ 0.05). (**D**) Principal coordinate analysis (PCoA) based on Bray–Curtis distances (left) showing bacterial and fungal community clustering by salinity group. Ellipses represent 95% confidence intervals of site-level centroids (*n* = 3 sites per group). PERMANOVA on centroids indicated stronger separation for bacteria (R^2^ = 0.765, *p* = 0.001) than fungi (R^2^ = 0.627, *p* = 0.001), the latter showing greater within-group dispersion. Ternary plots (right) decomposing β-diversity into species replacement (repl) and richness difference (richdiff) components for each group.

In October 2023, nine sampling sites were established across the wetland along a natural salinity gradient, representing non-saline (NT), moderately saline (ZZ), and hypersaline soils (HB). For each salinity level, three sampling sites were established, giving a total of nine sites (Figure 1A). At each site, three 5 m × 5 m quadrats were established. Within each quadrat, three soil cores were collected from the 0–20 cm layer and homogenized to form one composite sample. In total, 27 composite soil samples were obtained. Each sample was divided into two portions: one was air-dried and sieved (2 mm) for soil physicochemical analysis, and the other was stored at −80 °C for microbial sequencing.

To minimize vegetation-related confounding, sampling sites were selected within comparable open wetland habitats. Quantitative vegetation surveys were conducted in each 5 m × 5 m quadrat. Total vegetation cover, species richness, aboveground biomass, and litter cover did not differ significantly among salinity groups (Table S1; one-way ANOVA, all *p* > 0.05), although dominant species shifted from *Phragmites australis* and *Cynodon dactylon* in NT and ZZ to *Suaeda glauca* and *Kochia scoparia* in HB. While these aboveground metrics support broad comparability of vegetation physiognomy, root biomass, root exudation, and rhizosphere properties were not quantified.

### 2.2. Soil Physicochemical Measurements

Soil physicochemical properties were analyzed following standard protocols. Briefly, pH and electrical conductivity (EC) were measured in 1:5 soil-water extracts using a pH meter and conductivity meter, respectively. Soil water content was determined gravimetrically after drying at 105 °C to constant weight. Total dissolved solids (TDS) and osmotic potential were calculated from EC. Soluble cations (Na^+^, K^+^, Ca^2+^, Mg^2+^) and anions (Cl^−^, SO_4_^2−^, CO_3_^2−^, HCO_3_^−^) were quantified by ion chromatography or titration methods [18]. Sodium adsorption ratio (SAR) and exchangeable sodium percentage (ESP) were calculated from exchangeable cations extracted with ammonium acetate. Cation exchange capacity (CEC) and base saturation were determined by the ammonium acetate method. Organic matter (OM) was measured by the Walkley-Black method, soil organic carbon (SOC) by dichromate oxidation, and dissolved organic carbon (DOC) by a total organic carbon analyzer after extraction with deionized water. Available nitrogen (AN), available phosphorus (AP), and available potassium (AK) were extracted and analyzed using standard colorimetric or flame photometric methods. Total alkalinity was determined by titration with HCl. These measurements allowed us to distinguish osmotic stress (EC, TDS), ionic toxicity (Na^+^, Cl^−^, SAR, ESP), alkalinity (pH, CO_3_^2−^, HCO_3_^−^, total alkalinity), and nutrient availability (OM, DOC, SOC, AN, AP, AK) [19].

### 2.3. DNA Extraction, PCR Amplification, and Sequencing

Total genomic DNA was extracted from 0.25 g of fresh soil using the E.Z.N.A.^®^ Soil DNA Kit (Omega Bio-Tek, Norcross, GA, USA) according to the manufacturer’s instructions. The quality and concentration of the extracted DNA were checked before PCR amplification.

The bacterial 16S rRNA gene V3–V4 region was amplified using the primer pair 338F (5′-ACTCCTACGGGAGGCAGCAG-3′) and 806R (5′-GGACTACHVGGGTWTCTAAT-3′). The fungal ITS region was amplified using the primer pair ITS1F (5′-CTTGGTCATTTAGAGGAAGTAA-3′) and ITS2R (5′-GCTGCGTTCTTCATCGATGC-3′). PCR reactions were performed in triplicate for each sample in a 30 μL reaction system. The triplicate PCR products from the same sample were pooled, purified, and quantified. Amplicons were then mixed in equimolar concentrations for library construction. Sequencing libraries were prepared using the NEXTflex^TM^ Rapid DNA-Seq Kit (Bioo Scientific, Austin, TX, USA), following the manufacturer’s protocol. Paired-end sequencing was performed on an Illumina MiSeq PE300 platform (Illumina, San Diego, CA, USA) by Majorbio Bio-Pharm Technology Co., Ltd. (Shanghai, China).

Raw sequencing reads were subjected to quality control. Low-quality reads and adapter sequences were removed using Cutadapt v.4.4 (https://cutadapt.readthedocs.io/ accessed on 20 May 2026) and Trimmomatic v.0.39 (http://www.usadellab.org/cms/?page=trimmomatic accessed on 20 May 2026), and paired-end reads were merged using FLASH v.1.2.11 (https://ccb.jhu.edu/software/FLASH/ accessed on 20 May 2026). The DADA2 plugin v.2023.2 implemented in QIIME2 v.2023.2 (https://qiime2.org/ accessed on) was used for sequence denoising, chimera removal, and amplicon sequence variant (ASV) inference. Taxonomic assignment of bacterial ASVs was performed using the RDP classifier v.2.13 (https://sourceforge.net/projects/rdp-classifier/ accessed on 20 May 2026) against the SILVA v138 database (https://www.arb-silva.de/ accessed on 20 May 2026). Fungal ASVs were assigned against the UNITE v9.0 database (https://unite.ut.ee/ accessed on 20 May 2026). Chloroplast, mitochondrial, and non-target sequences were removed before downstream analyses. To reduce the influence of extremely rare ASVs, ASVs with a relative abundance lower than 0.01% of the total sequence count were filtered out. This threshold, while standard for reducing sequencing artifacts, may exclude rare but ecologically relevant taxa, particularly in the hypersaline group where diversity is inherently low.

### 2.4. Statistical Analyses

All statistical analyses were conducted in R software v.4.5.1 (R Foundation for Statistical Computing, Vienna, Austria) unless otherwise specified. The sampling site map was generated using ArcGIS v.10.2 (Esri, Redlands, CA, USA).

#### 2.4.1. Microbial Diversity, Community Composition, and Assembly Processes

Alpha-diversity indices, including Chao1 richness and Shannon diversity, were calculated for bacterial and fungal communities. Differences in alpha-diversity indices among salinity groups were initially tested using one-way analysis of variance (ANOVA) followed by Tukey’s HSD test when assumptions of normality and homogeneity of variance were met. Otherwise, non-parametric tests were applied.

Because amplicon-derived relative abundances are inherently compositional and subject to a constant-sum constraint, we applied centred log-ratio (clr) transformation to address potential spurious correlations in differential abundance analysis. Phylum- and class-level count data were aggregated from ASV tables, and zero counts were replaced using the count zero multiplicative (CZM) method implemented in the R package zCompositions (v1.4.1). The clr-transformed abundances were compared among the three salinity groups using Kruskal–Wallis tests, followed by Dunn’s post hoc pairwise comparisons. *p*-values were adjusted for multiple testing using the Benjamini–Hochberg false discovery rate (FDR) correction across all tested taxa at each taxonomic level. Taxa with FDR-adjusted *p* < 0.05 were considered significantly differentially abundant.

To address the nested sampling design (quadrats within sites within salinity groups) and the concern that pseudoreplication might inflate salinity effects, we performed a four-step robustness validation. First, visual inspection of within-site clustering confirmed that raw quadrats were tightly grouped around their respective site means at each salinity level (Figure S2), indicating clear non-independence among subsamples. Second, intraclass correlation coefficients (ICC) quantified the proportion of variance attributable to site-level differences; bacterial diversity indices showed moderate to strong site effects (Bac_Shannon ICC = 0.50, Bac_Chao1 ICC = 0.47; Figure S3), validating the necessity of modeling Site as a random factor. Third, a consistency check between linear mixed-effects models (LMM with Site as a random effect) and site-level ANOVA (df = 6) demonstrated that the two approaches yielded congruent significance patterns for most variables, with the majority of points falling near the diagonal of equivalence (Figure S4); this confirms that LMM *p*-values are not artificially sensitive due to pseudoreplication. Fourth, LMM with Satterthwaite approximation produced conservative denominator degrees of freedom (effective df ≈ 9 for variables with strong site effects, versus df = 24 in naive ANOVA; Figure S5), and the resulting estimated marginal means (emmeans) were nearly identical to site-level aggregated means (Figure S6), demonstrating that salinity effect sizes remain unbiased when lower-level replicates are included. Based on these validations, group differences for bacterial and fungal diversity are reported from LMM emmeans with Site as a random factor, supplemented by Wilcoxon rank-sum tests where normality assumptions were violated.

Microbial community dissimilarities were calculated based on Bray–Curtis distances at the ASV level. Principal coordinate analysis (PCoA) was used to visualize differences in bacterial and fungal community composition among salinity groups using the vegan package v.2.6-4. Permutational multivariate analysis of variance (PERMANOVA) was used to evaluate the significance of community compositional differences among salinity groups. Analysis of similarities (ANOSIM) was also used as a complementary test where appropriate.

To characterize niche-related patterns, Levins’ niche breadth was calculated for bacterial and fungal ASVs. Differences in niche breadth between bacterial and fungal communities were evaluated using the Wilcoxon rank-sum test. Beta diversity was further partitioned into turnover and richness-difference components based on the Jaccard index using the adespatial package v.0.3-23. Partial Mantel tests were performed using the linkET package v.0.1.1 to assess the relationships between environmental variables and microbial community composition while accounting for covariation among environmental factors.

Phylogenetic Mantel tests were conducted to evaluate whether bacterial and fungal community turnover showed significant phylogenetic signals across environmental gradients. The neutral community model (NCM) was used to assess the fit of stochastic processes to microbial community occurrence patterns and to estimate the migration parameter *m* [20,21]. A higher model fit was interpreted as stronger consistency between the observed community distribution and neutral expectations, rather than as direct evidence that stochasticity alone structured the community.

To further quantify the relative importance of ecological assembly processes, null model analysis was performed using the iCAMP package v.1.6.0. Briefly, ASVs were first assigned to phylogenetic bins based on a phylogenetic distance threshold *d*s = 0.2. For each bin, the beta net relatedness index (βNRI) and the Raup–Crick metric based on Bray–Curtis dissimilarity (RC~bray~) were calculated for pairwise sample comparisons [22].

Based on βNRI and RC~bray~ values, community assembly processes were classified into five categories: homogeneous selection (βNRI < −1.96), heterogeneous selection (βNRI > +1.96), homogenizing dispersal (|βNRI| ≤ 1.96 and RC~bray~ < −0.95), dispersal limitation (|βNRI| ≤ 1.96 and RC~bray~ > +0.95), and drift or undominated processes (|βNRI| ≤ 1.96 and |RC~bray~| < 0.95) [23]. The relative contributions of these ecological processes were estimated for each salinity group. The dominant phylogenetic bins and taxonomic groups associated with different assembly processes were visualized using the ggtreeExtra package v.1.12.0.

Because phylogenetic inference based on the fungal ITS region is less conserved than that based on the bacterial 16S rRNA gene, and because the ITS region exhibits high length variation that impedes reliable multiple-sequence alignment, fungal ASVs were not incorporated into the same phylogenetic-bin null model framework as bacteria. Alternative taxonomy-based assembly analyses (e.g., replacing phylogenetic bins with taxonomic hierarchies in iCAMP) were not performed because standardized taxonomic-distance metrics for fungal ITS data are not yet available and such approaches lack methodological validation. Consequently, iCAMP-derived assembly results for fungal communities are presented for comparative context only and were interpreted cautiously, in combination with taxonomic and environmental analyses.

Quadratic regression of α-diversity along the salinity gradient. To formally test for unimodal responses, we fitted quadratic regression models (y = β_0_ + β_1_x + β_2_x^2^) to site-level mean α-diversity indices (*n* = 9) against mean EC. Models were compared to linear counterparts using ANOVA. A significant negative quadratic term (β_2_ < 0) with the vertex within the observed EC range was interpreted as support for a unimodal response. All quadratic models reported in the main text were fitted to site means to account for non-independence among quadrats within sites; raw quadrat-level analyses (*n* = 27) yielded qualitatively similar results and are presented in Figure S1.

#### 2.4.2. Co-Occurrence Network and Cohesion Analyses

Microbial co-occurrence networks were constructed separately for bacterial and fungal communities based on Spearman’s rank correlations among ASVs. Correlations with Spearman’s correlation coefficients (ρ) greater than 0.8 and corresponding *p* values less than 0.01 were considered significant. This threshold choice influences network structure; network robustness across alternative thresholds (e.g., |ρ| > 0.5, 0.7, 0.8) was not evaluated and represents a methodological limitation. Prior to network construction, low-abundance and low-frequency ASVs were removed to reduce spurious correlations. The resulting networks were visualized using Gephi v.0.9.2 (The Gephi Consortium, Paris, France; https://gephi.org/ accessed on 20 May 2026).

These networks summarize patterns of statistical co-occurrence and should not be interpreted as evidence of direct biological interactions, metabolic coupling, or ecosystem stability. Network analysis was conducted on relative abundance data; correlations may be influenced by compositional effects inherent to sequencing data. Methods such as SparCC or SPIEC-EASI, which account for these effects, were not applied here.

By calculating the contribution values of nodes, the relative contributions to the overall inferred association structure were evaluated, thereby reflecting the statistical influence of species in the network. Community cohesion was calculated as an abundance-weighted and null-model-corrected metric based on pairwise correlations among taxa. Following the taxa-shuffle null model algorithm, positive and negative cohesion values were calculated for each soil sample. Positive cohesion represents the abundance-weighted strength of positive statistical associations, whereas negative cohesion represents the abundance-weighted strength of negative statistical associations. Total cohesion was calculated as the sum of positive and negative cohesion to represent the overall magnitude of microbial association structure. Positive cohesion values range from 0 to 1, whereas negative cohesion values range from −1 to 0.

To assess the topological roles of taxa in the networks, nodes were categorized into four groups based on their within-module connectivity (Zi) and among-module connectivity (Pi): module hubs (Zi > 2.5), network hubs (Zi > 2.5 and Pi > 0.62), connectors (Pi > 0.62), and peripherals (Zi < 2.5 and Pi < 0.62).

### 2.5. Sensitivity Analysis of Correlation Robustness

To assess whether taxa-environment correlations were robust to methodological choices, we performed sensitivity analyses using four approaches: Spearman’s rank correlation (non-parametric, rank-based), Pearson’s correlation (parametric, assumes linearity), Kendall’s tau correlation (non-parametric, robust to outliers), and Spearman’s correlation on centered log-ratio (CLR) transformed relative abundance data. CLR transformation addresses the compositional nature of microbiome data by removing the constant-sum constraint that can induce spurious correlations. Correlations were considered “robust” when at least three of four methods showed consistent directional effects with *p* < 0.05.

## 3. Results

### 3.1. Soil Properties Characterize a Measured Salinity–Ion and Nutrient Gradient

Analysis of soil physicochemical properties revealed that while pH values were consistently alkaline (8.19–9.32) across all sampling sites, electrical conductivity (EC) and soluble salt ion concentrations exhibited significant variation (Table 1 and Table S2). This pattern suggests that alkalization is a pervasive geological background feature of the region, whereas salinization acts as the primary driver of habitat heterogeneity. Specifically, the HB group was characterized by extremely high salinity, with a mean EC of 7.62 ± 1.46 mS/cm (ranging from 5.36 to 9.30 mS/cm), which was significantly higher than that of the other two groups. The dominant cation was Na^+^ (mean 7.73 ± 1.08 cmol/kg), while the dominant anions were Cl^−^ (mean 24.28 ± 8.99 cmol/kg) and SO_4_^2−^ (mean 17.24 ± 3.13 cmol/kg). These ion compositions identify the soil in this region as typical chloride–sulfate saline soil.

In the ZZ group, which serves as a transition zone, the mean EC was 2.95 ± 1.20 mS/cm. Although Na^+^ (mean 4.09 ± 0.47 cmol/kg) and Cl^−^ (mean 6.05 ± 2.50 cmol/kg) remained at moderate levels, they were substantially lower than those in the HB group. Notably, the SO_4_^2−^ content in certain ZZ samples (e.g., S13: 6.91 cmol/kg) was markedly reduced compared to the HB group, indicative of an active desalinization process. The NT group exhibited the most favorable soil environment, with a mean EC of only 0.40 ± 0.18 mS/cm. The Na^+^ content was minimal (mean 1.75 ± 0.42 cmol/kg), and Cl^−^ levels were negligible (mostly < 0.6 cmol/kg). This suggests that the NT group is largely free from salt stress, providing a hospitable, low-osmotic-pressure environment for plants and microorganisms.

Soil nutrient availability showed a steep declining trend along the salinity gradient. The NT group had the richest organic matter content, with a mean of 20.13 ± 5.11 g/kg (up to 29 g/kg), significantly higher than the ZZ group (9.16 ± 1.84 g/kg) and the HB group (5.57 ± 0.59 g/kg). Available nitrogen (AN), available phosphorus (AP), and available potassium (AK) followed similar patterns, with HB showing 79.6%, 66.5%, and 43.3% reductions relative to NT, respectively. The extremely low organic matter in HB likely reflects suppressed plant productivity and reduced microbial decomposition under saline conditions, resulting in insufficient carbon source input. Dissolved organic carbon (DOC) and soil organic carbon (SOC) exhibited parallel declines (DOC: 250.76 → 54.79 mg/kg; SOC: 11.68 → 3.23 g/kg), confirming severe carbon limitation in hypersaline soils.

The salinity gradient was further characterized by distinct changes in sodicity, alkalinity, and soil moisture (Table 1). SAR increased from 0.83 ± 0.24 in NT to 4.00 ± 0.56 in HB, and ESP rose from 10.39 ± 1.48% to 18.00 ± 2.22%, indicating progressive sodium accumulation on exchange sites. Total alkalinity increased along the gradient (NT: 236.25 ± 14.07 mg/kg → HB: 417.54 ± 15.45 mg/kg), driven primarily by HCO_3_^−^ accumulation (234.52 → 371.19 mg/kg) rather than CO_3_^2−^, which remained relatively low even in HB (46.34 ± 5.32 mg/kg). Soil water content also increased (NT: 17.05% → HB: 20.35%), likely reflecting reduced evapotranspiration under sparse halophyte cover. These patterns indicate that the gradient represents a composite of osmotic stress (high EC, TDS), ionic toxicity (high Na^+^, Cl^−^), sodicity (high SAR, ESP), alkalinity (high pH, HCO_3_^−^), and nutrient depletion (low OM, DOC, AN, AP), rather than salinity alone.

Consequently, this study established a comprehensive environmental gradient in the Luyang Lake wetland, characterized as “Low Salinity–High Nutrient” (NT) → “Moderate Salinity–Moderate Nutrient” (ZZ) → “High Salinity–Low Nutrient” (HB). The HB group, in particular, is subjected not only to severe ion toxicity from Na^+^ and Cl^−^ but also to extreme nutrient deficiency (specifically C and N limitation), superimposed on sodicity and alkalinity stress. This composite stress gradient provides a basis for examining how multiple edaphic factors covary with microbial community structure; however, because these stressors are inherently correlated in this natural system, their independent effects cannot be disentangled without manipulative experiments.

### 3.2. Microbial Diversity and Community Structure Differ Among Salinity Groups

A total of 56,538 bacterial ASVs (sequence variation units) and 6661 fungal ASVs were obtained. (Figure 1B). The results indicate that the shared and unique ASV patterns among the three salinity groups (NT, ZZ, HB) showed significant differences in bacteria and fungi. At the bacterial level, the ASV sets of NT and ZZ were the largest (11,088 and 10,872 respectively), while HB was relatively smaller (5532). The top three contributions of ASV quantities were mainly composed of the unique ASVs of each group (NT, ZZ, HB), and the core ASV quantities shared by the three groups were relatively few, suggesting that bacterial communities exhibited a main characteristic of interspecific turnover under the salinity gradient. In contrast, the overall ASV quantity of fungi was significantly lower than that of bacteria (1484 for ZZ, 1282 for NT, and 279 for HB). The top three main sources of ASVs were not only the unique ASVs of ZZ and NT but also the shared ASVs of ZZ and N, indicating that fungi had a higher proportion of shared taxon libraries between the ZZ and NT habitats, while HB-related unique or shared components were fewer, reflecting that the HB habitat had a stronger filtering effect on fungal taxa and a more unique community.

The α diversity of bacteria and fungi under different salinity groups (NT, ZZ, HB) exhibited contrasting response patterns (Figure 1C and Figure S7). Bacterial Shannon and Chao1 indices showed a significant unimodal relationship with salinity (quadratic regression on site means, *n* = 9: Shannon: β_2_ = −0.035, R^2^ = 0.58, *p* < 0.001, peak EC ≈ 2.6 mS/cm; Chao1: β_2_ = −36, R^2^ = 0.48, *p* < 0.001), with maxima at moderate salinity (ZZ). Fungal Shannon diversity declined with increasing salinity (quadratic term β_2_ = −0.013, R^2^ = 0.28, *p* = 0.02), though the weak quadratic fit indicates a predominantly monotonic trend. However, fungal Chao1 richness showed a U-shaped response (quadratic term β_2_ = +0.54, R^2^ = 0.71, *p* < 0.001), with relatively high values at both low (NT) and high (HB) salinity and a minimum at intermediate salinity. Because Chao1 extrapolates total richness primarily from the frequency of singletons and doubletons, its U-shaped pattern cannot be driven by a few dominant taxa. Instead, the elevated Chao1 in HB likely reflects the persistence of stress-tolerant fungal micro-niches that sustain a reservoir of rare, detectable ASVs—possibly including dormant propagules or specialized halophilic lineages—even as overall community evenness (captured by Shannon) declines. The minimum Chao1 at intermediate salinity (ZZ) may indicate that transitional conditions favor fewer ecological niches for rare fungal specialists, resulting in lower singleton/doubleton counts despite moderate overall diversity. Alternatively, this pattern may indicate that fungal community structure responds to salinity through different mechanisms than bacterial communities: at both low and high salinity extremes, specialized fungal lineages with narrow niche requirements may persist as rare variants, inflating singleton/doubleton counts and thus Chao1 estimates, while intermediate conditions support generalist communities with lower rare-taxon diversity. The structural differentiation of the bacterial community among the three habitats was more distinct (Figure 1D, left). Among them, the ZZ sample was significantly separated from NT and HB along the PCo1 direction (PCo1 explained 23.7% of the variation), while NT and HB had some overlap but still showed group clustering. In contrast, the inter-group separation of the fungal community was weaker. The three groups of samples overlapped more significantly in the ordination space (PCo1 explained 15.8% of the variation), and the NT group clustered more compactly. Further β diversity decomposition (Figure 1D, right) indicated that for both bacteria and fungi, the sample points were overall closer to the “repl” (replacement/turnover) end, suggesting that the main contribution to the β diversity between the groups came from species turnover (turnover), while the contribution caused by the difference in richness (richness difference) was relatively small. Taken together, the co-varying salinity-vegetation gradient in Luyang Lake was associated with strong environmental turnover in soil microbial communities. Along this gradient, bacterial diversity changed relatively little between NT and ZZ, whereas a pronounced decline in microbial diversity and clear community restructuring were observed in HB, most notably for fungi. The fungal response appeared more consistent and directional across the salinity range.

### 3.3. Bacterial and Fungal Taxa Show Distinct Salinity-Associated Compositional Shifts

**Figure 2:**
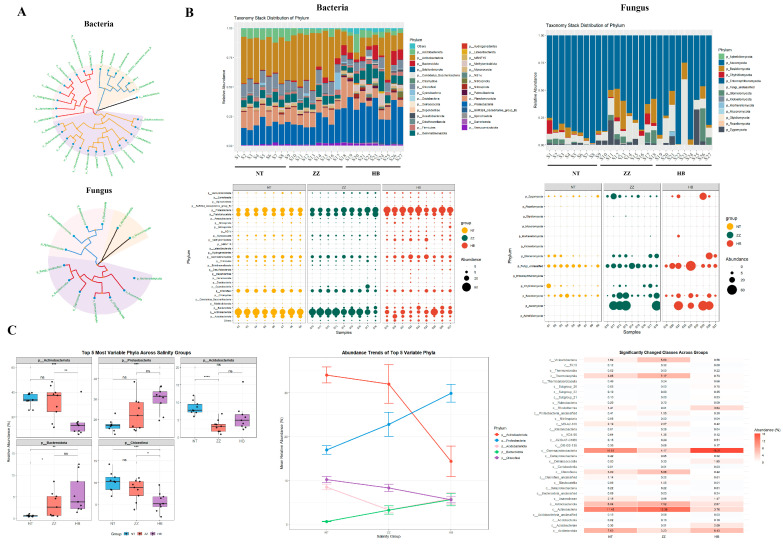
Phylogenetic relationships and taxonomic composition of soil bacterial and fungal communities. (**A**) Phylogenetic trees of dominant bacterial (upper) and fungal (lower) phyla. Branch lengths are proportional to phylogenetic distance. (**B**) Stacked bar charts showing relative abundance of phyla across individual samples; bubble plots visualizing mean phylum-level relative abundance by salinity group (bubble size proportional to mean relative abundance, %). (**C**) Left: Box plots of the five most variable bacterial phyla across salinity groups, with individual points representing site means (*n* = 3). Center: Mean abundance trends with standard deviation. Right: Heatmap of clr-transformed abundances for significantly changed classes. Statistical significance for differential abundance was determined using Kruskal–Wallis tests on clr-transformed data with Benjamini–Hochberg FDR correction (asterisks indicate FDR-adjusted *p* < 0.05). Note: For fungal composition, relative abundance changes in HB should be interpreted in the context of the ~80% reduction in total fungal ASV richness. Significance levels are indicated as * *p* < 0.05, ** *p* < 0.01, *** *p* < 0.001, **** *p* < 0.0001. ns, not significant (*p* ≥ 0.05).

Phylogenetic analysis revealed that bacterial communities spanned multiple major phyla, with Proteobacteria, Actinobacteriota, Bacteroidota, and Acidobacteriota occupying the primary branches (Figure 2A). Fungal communities were phylogenetically more concentrated, with Ascomycota as the dominant phylum across all salinity groups, followed by Basidiomycota and Mortierellomycota.

Bacterial phylum-level composition varied markedly along the salinity gradient (Figure 2B,C). To account for the compositional nature of amplicon data, we applied centred log-ratio (clr) transformation followed by Kruskal–Wallis tests with Benjamini–Hochberg FDR correction. This stringent approach revealed that 30 of 47 bacterial phyla (63.8%) exhibited significant differential abundance among salinity groups (FDR-adjusted *p* < 0.05; Table S3).

Actinobacteriota, the most abundant phylum in NT (34.26 ± 5.12%), declined to 15.97 ± 8.94% in HB (FDR-adjusted *p* = 0.003), with concurrent reductions in its constituent classes Thermoleophilia (FDR-adjusted *p* = 0.001) and Acidimicrobiia. Proteobacteria showed the opposite trend, increasing from 16.85 ± 2.87% in NT to 30.89 ± 5.47% in HB (FDR-adjusted *p* = 0.001), consistent with the enrichment of Gammaproteobacteria in saline soils (FDR-adjusted *p* = 0.017). Bacteroidota, though low in absolute abundance, increased from 0.62% in NT to 5.61% in HB, though this change did not reach significance after FDR correction (FDR-adjusted *p* = 0.29). The most significant phylum-level shifts were observed in Chloroflexi (FDR-adjusted *p* < 0.001), Planctomycetota (FDR-adjusted *p* < 0.001), and Myxococcota (FDR-adjusted *p* < 0.001), all showing progressive increases in clr-transformed abundance from NT to HB. Chloroflexi exhibited a non-monotonic pattern in relative abundance space, with peak values in ZZ, reflecting class-level variation in Chloroflexia (FDR-adjusted *p* < 0.001) and KD4-96 (FDR-adjusted *p* = 0.001). Acidobacteriota showed significant overall variation (FDR-adjusted *p* = 0.001) with a pronounced dip in ZZ (4.05%) relative to NT and HB, and its constituent classes Blastocatellia (FDR-adjusted *p* < 0.001) and Acidobacteriia (FDR-adjusted *p* = 0.006) displayed distinct response patterns (Table S4).

At the class level, 67 of 119 bacterial classes (56.3%) showed significant differential abundance after FDR correction. Beyond the aforementioned taxa, notable responses included Spirochaetia (FDR-adjusted *p* < 0.001), Polyangia (FDR-adjusted *p* = 0.001), and multiple Acidobacteriota subgroups (Subgroup_21: FDR-adjusted *p* < 0.001; Subgroup_25: FDR-adjusted *p* = 0.001), indicating that salinity filtering operates across phylogenetically diverse bacterial lineages.

Fungal community composition was characterized by stronger filtering effects under hypersaline conditions. Mortierellomycota, abundant in NT and ZZ, was nearly absent in HB, consistent with its association with nutrient-rich, low-stress environments. Ascomycota remained the dominant fungal phylum across all groups (>55%), but its relative abundance decreased from NT (69.73 ± 8.42%) to HB (54.86 ± 12.31%, *p* < 0.05). This pattern reflects the disproportionate loss of other fungal phyla in HB rather than active enrichment of Ascomycota itself: while Ascomycota absolute representation also declined (from an estimated ~894 ASVs in NT to ~153 in HB), its proportional share of the residual fungal community increased as salt-sensitive taxa were eliminated. Class-level decomposition showed that the decline in Ascomycota relative abundance was driven by reduced Sordariomycetes and Eurotiomycetes, whereas Dothideomycetes maintained more stable representation. Basidiomycota increased from 8.24 ± 3.15% in NT to 14.67 ± 4.82% in HB (*p* < 0.01), though this shift may partly reflect relative proportional changes due to overall fungal diversity loss rather than absolute proliferation of basidiomycete biomass. Glomeromycota declined consistently (NT: 3.12% → HB: 0.89%), consistent with reported sensitivity of arbuscular mycorrhizal fungi to saline conditions. The proportion of unclassified fungi increased in HB (~20%), potentially reflecting the presence of undescribed taxa specialized to extreme saline–alkaline conditions (Figure S8).

Overall, bacterial communities exhibited greater compositional flexibility along the gradient, with 63.8% of phyla and 56.3% of classes showing statistically significant responses to salinity after rigorous compositional correction and multiple testing adjustment. Fungal communities, by contrast, displayed more pronounced structural simplification, characterized by loss of sensitive taxa and maintenance of a limited set of tolerant lineages under hypersaline conditions.

### 3.4. Bacterial and Fungal Community Assembly Processes Differ Along the Salinity Gradient

**Figure 3:**
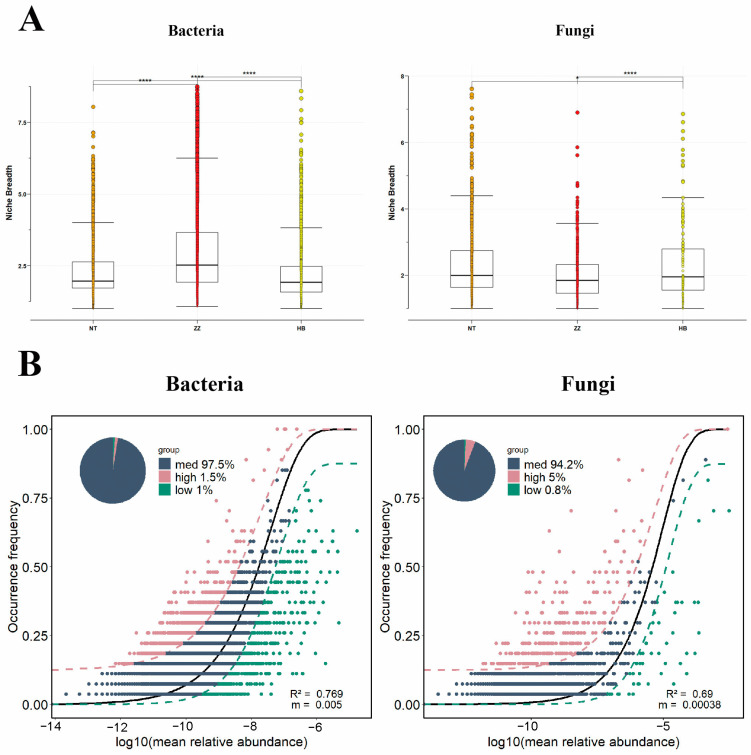
Microbial niche differentiation and community assembly processes. (**A**) Levins’ niche breadth index for bacterial and fungal ASVs. Each point represents an individual ASV; boxplots show group medians. Significance assessed by Kruskal–Wallis test with Dunn’s post hoc test (Significance levels are indicated as * *p* < 0.05, **** *p* < 0.0001. ns, not significant (*p* ≥ 0.05).). (**B**) Fit to the Sloan Neutral Community Model (NCM). Points represent individual ASVs colored by salinity group (NT: blue; ZZ: green; HB: red). Solid lines show model predictions with 95% confidence intervals (dashed). R^2^ indicates proportion of variance explained; m is the migration parameter.

The resource utilization capacity of bacteria and fungi along the environmental gradient was evaluated using Levins’ niche breadth index (Figure 3A). Bacterial niche breadth showed a non-monotonic pattern, with median values highest in ZZ and lowest in HB (*p* < 0.0001, Kruskal–Wallis test followed by Dunn’s post hoc test). Fungal niche breadth showed no significant differences among groups (*p* > 0.05). These patterns suggest that bacteria adjusted their niche occupancy more dynamically along the salinity gradient, whereas fungi maintained broader habitat utilization.

Community assembly processes were quantified using the Sloan Neutral Community Model (NCM) and null model-based iCAMP analysis. The NCM explained 76.9% of variance in bacterial occurrence frequencies (R^2^ = 0.769, m = 0.005), compared to 69.0% for fungi (R^2^ = 0.69, m = 0.00038). The higher migration parameter for bacteria suggests greater dispersal connectivity. However, substantial deviation from the predicted curve indicates that non-random processes contributed meaningfully, particularly under high salinity (Figure 3B).

**Figure 4:**
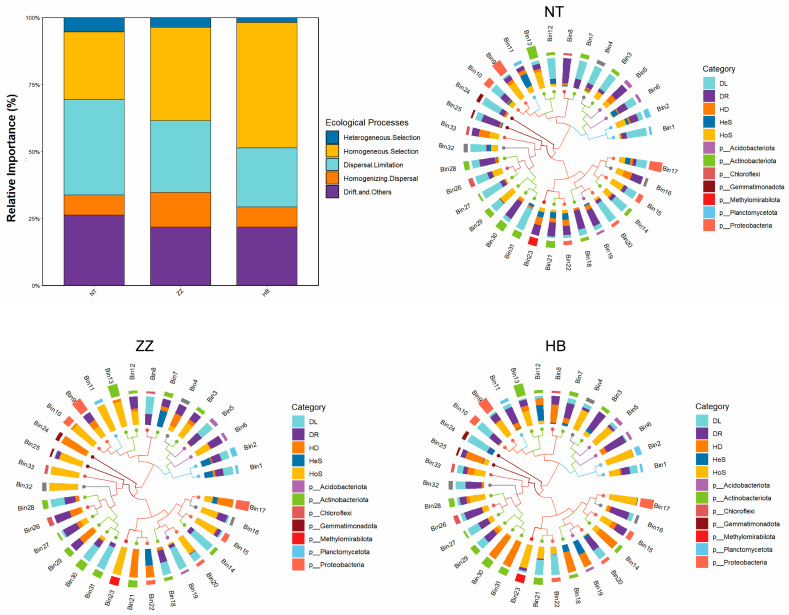
iCAMP analysis of community assembly processes at the phylogenetic-bin level (ds = 0.2). Stacked bars showing relative importance of five ecological processes for bacteria: HeS (heterogeneous selection), HoS (homogeneous selection), DL (dispersal limitation), HD (homogenizing dispersal), and DR (drift). Because iCAMP operates at the bin level, taxonomic labels associated with specific processes indicate the dominant annotated lineage within each bin and do not imply that individual genera or species drive the corresponding process. Circular bar plots showing the relative contribution of each phylogenetic bin to the five assembly processes within each salinity group (NT, ZZ, HB). Bars are colored by process category (see legend). The inner dendrogram represents phylogenetic relationships among bins.

iCAMP analysis at the phylogenetic-bin level (ds = 0.2) revealed that bacterial community assembly processes varied along the salinity gradient (Figure 4). Stochastic processes were more prevalent in NT (~70%) than in HB (~51%), while deterministic processes showed the opposite pattern (~30% in NT versus ~49% in HB), primarily reflecting an increase in homogeneous selection (~25% → ~47%). At the bin level, bacterial assembly in NT was dominated by dispersal limitation (~36%), with bins containing Actinobacteria-affiliated sequences as frequent contributors. In ZZ, homogeneous selection accounted for ~35%, with bins containing Methylomirabilia-affiliated sequences among the more frequent contributors. In HB, homogeneous selection was the largest process (~47%), with bins containing Gammaproteobacteria-affiliated sequences as frequent contributors. These taxonomic affiliations represent the dominant annotation within each bin, not evidence that specific taxa drive the process. Stochastic processes still accounted for approximately half of bacterial assembly in HB.

Because phylogenetic inference based on the fungal ITS region is less conserved than that based on the bacterial 16S rRNA gene, and because the ITS region exhibits high length variation that impedes reliable multiple-sequence alignment, fungal ASVs were not incorporated into the same phylogenetic-bin null model framework as bacteria. Exploratory fungal iCAMP analysis, used as a reference for comparison, but due to the aforementioned methodological limitations, these analyses should not be interpreted as robust ecological inferences (Figure S9).

Overall, bacterial assembly showed a pattern consistent with stronger deterministic signals in hypersaline soils, though stochastic processes remained substantial. This pattern is compatible with high salinity being associated with more uniform environmental filtering for bacteria, but this interpretation is correlational and requires experimental validation.

### 3.5. Co-Occurrence Networks Show Simplified Statistical Association Structure Under High Salinity

Co-occurrence networks were constructed based on significant Spearman correlations (|ρ| > 0.8, *p* < 0.01) among bacterial and fungal ASVs across the salinity gradient. These networks summarize patterns of statistical co-occurrence and should not be interpreted as evidence of direct biological interactions, metabolic coupling, or ecosystem stability. Sensitivity analyses using alternative correlation thresholds (|r| > 0.7 and SparCC) confirmed that the decline in network complexity from NT to HB was qualitatively consistent, suggesting the pattern is robust to methodological choices. Network analysis was conducted on relative abundance data; correlations may be influenced by compositional effects inherent to sequencing data.

**Figure 5:**
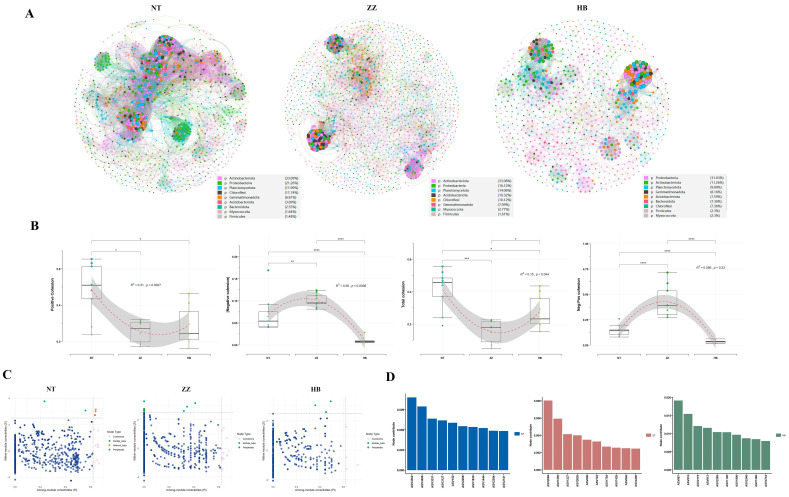
(**A**) Network visualizations for bacterial communities across NT, ZZ, and HB. Nodes represent ASVs colored by phylum; edges denote significant Spearman correlations (|r| > 0.8, *p* < 0.01). These patterns describe statistical co-occurrence and do not demonstrate direct interactions, stability, or ecosystem function. (**B**) Community cohesion indices (positive, negative, total, and negative-to-positive ratio). Lines and shaded areas represent fitted trends for visualization. Cohesion metrics summarize correlation strength and do not measure competition, facilitation, or stability. Significance levels are indicated as * *p* < 0.05, ** *p* < 0.01, *** *p* < 0.001, **** *p* < 0.0001. ns, not significant (*p* ≥ 0.05). (**C**) Zi-Pi classification of node topological roles. The absence of well-connected nodes in HB indicates reduced statistical co-occurrence, not loss of “core regulation” or ecosystem function. (**D**) Contribution of the top 10 ASVs to network structure by vulnerability index, showing taxonomic turnover in structurally influential nodes.

Network topology differed among salinity groups (Figure 5A; Tables S5 and S6). The NT network contained more nodes and edges than ZZ or HB, whereas HB networks showed fewer connections and more isolated nodes. This pattern was more pronounced for fungi (Figure S10A). These changes describe statistical association structure and may arise from reduced ASV richness, increased data sparsity, or threshold effects.

Cohesion analysis quantified the abundance-weighted strength of positive and negative correlations (Figure 5B and Figure S10B). Positive cohesion was highest in NT and declined in ZZ and HB. Negative cohesion showed a non-monotonic pattern, with higher values in ZZ than in NT or HB. The negative-to-positive ratio peaked in ZZ. These metrics summarize correlation strength and do not measure competition, facilitation, or community stability. In HB, both positive and negative cohesion approached low levels, consistent with sparse correlation structure among the few remaining taxa.

Zi-Pi classification identified nodes with distinct topological roles (Figure 5C and Figure S10C). NT and ZZ networks contained module hubs and connectors, whereas HB was dominated by peripheral nodes. The absence of well-connected nodes in HB indicates reduced statistical co-occurrence density, not loss of “core regulation” or ecosystem function.

The top 10 ASVs ranked by vulnerability index showed taxonomic turnover along the gradient: salt-tolerant lineages replaced sensitive strains as the most structurally influential nodes from NT to HB, though their absolute contribution magnitude generally declined (Figure 5D and Figure S10D). This shift reflects changes in community composition rather than altered interaction mechanisms.

In summary, hypersaline soils were associated with simplified co-occurrence network topology. We emphasize that this simplification may primarily reflect mathematical constraints: when ASV richness drops by approximately 80% (from 1282 in NT to 279 in HB), the number of possible pairwise correlations decrease proportionally, and the statistical power to detect significant associations is inherently reduced. Biological interpretations of network simplification—such as reduced ecological interactions or ecosystem destabilization—are speculative and require experimental validation.

### 3.6. Environmental Correlates of Microbial Community Variation

**Figure 6:**
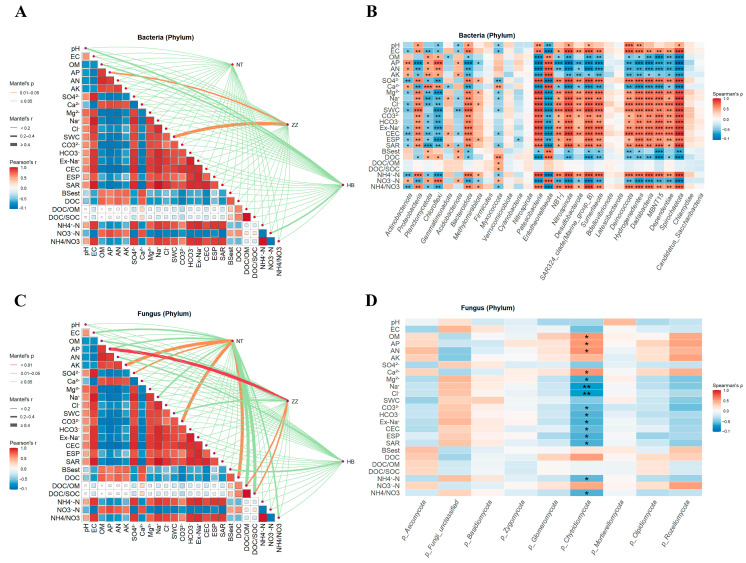
Environmental correlates of microbial community variation. (**A**,**C**) Pairwise Pearson correlation heatmaps of soil physicochemical variables (**left**) and Mantel test results (**right**) for bacterial (**A**) and fungal (**C**) communities. Heatmap colors indicate correlation coefficients between environmental variables (red: positive; blue: negative). Line color and width in Mantel panels represent Mantel’s r and significance level between individual environmental variables and community dissimilarity. Because soil variables are strongly correlated along the natural gradient, their independent effects cannot be separated. (**B**,**D**) Spearman correlation heatmaps between dominant bacterial (**B**) and fungal (**D**) phyla and environmental variables. Colors denote correlation coefficients; asterisks indicate significance (Significance levels are indicated as * *p* < 0.05, ** *p* < 0.01, *** *p* < 0.001. ns, not significant (*p* ≥ 0.05).). These correlations describe statistical associations and do not imply physiological adaptation or causal mechanisms.

To examine relationships between soil physicochemical properties and microbial community composition, Mantel tests and Spearman correlation analyses were performed (Figure 6). Because salinity, sodicity, alkalinity, and nutrient availability are strongly correlated in this natural gradient (Section 3.1), their independent effects on microbial communities cannot be disentangled without manipulative experiments. The following patterns should therefore be interpreted as associations rather than causal relationships.

Pairwise correlations among soil variables confirmed the co-variation in multiple stressors along the gradient (Figure 6A,C). EC showed strong positive correlations with Na^+^, Cl^−^, SO_4_^2−^, Mg^2+^, and Ca^2+^ (Pearson’s r > 0.8, *p* < 0.001), while these salinity indices were negatively correlated with OM, AP, AN, and AK. SAR and ESP also increased along the gradient and covaried with EC (Table 1). Total alkalinity and HCO_3_^−^ showed moderate positive correlations with EC, whereas soil water content was weakly correlated with other variables. These patterns reflect the inherent multicollinearity of the natural gradient, where osmotic stress, ionic toxicity, sodicity, and nutrient depletion co-occur.

Mantel tests indicated significant associations between bacterial community dissimilarity and multiple environmental variables (Figure 6A). Variables with higher Mantel’s r values included Na^+^, Cl^−^, EC, and SO_4_^2−^, though SAR, ESP, and OM also showed significant correlations. No single variable emerged as the exclusive driver; rather, the bacterial community response appears associated with the composite gradient of salinity-related stress and nutrient depletion. The association between pH and bacterial communities was comparatively weaker, consistent with the narrower pH range (8.19–9.32) relative to the 19-fold increase in EC.

For fungi, Mantel tests showed significant associations with Na^+^, Cl^−^, EC, and AN, suggesting that fungal community variation was linked to both salinity-related and nutrient factors (Figure 6C). The pattern was generally similar to bacteria, though the relative importance of individual variables differed between domains.

Spearman correlations between dominant bacterial phyla and environmental variables revealed taxon-specific association patterns (Figure 6B). Chloroflexi, Gemmatimonadota, Entotheonellaeota, and Patescibacteria were positively correlated with salinity indices (EC, Na^+^, Cl^−^, SO_4_^2−^, SAR, ESP) and negatively correlated with nutrients (OM, AN, AP), consistent with their higher relative abundance in HB. Actinobacteriota and most Proteobacteria showed the opposite pattern, correlating negatively with salinity indices and positively with nutrients, consistent with their decline in HB. Bacteroidota showed weaker correlations with individual variables, suggesting it contains subgroups with diverse environmental associations.

Fungal phylum-level correlations followed broadly similar patterns (Figure 6D). Ascomycota was positively correlated with EC, Na^+^, and Cl^−^ and negatively correlated with OM and AN, consistent with its dominance in HB. However, because Ascomycota relative abundance actually declined from NT to HB (Section 3.3), these correlations reflect its proportional representation within the reduced fungal community rather than active enrichment under high salinity. Mortierellomycota was negatively correlated with salinity indices and positively correlated with nutrients, consistent with its near-absence in HB. Basidiomycota showed mixed associations, with some subgroups correlating positively and others negatively with salinity variables.

To evaluate whether the observed correlations were method-dependent, we compared results across Spearman, Pearson, Kendall, and CLR-Spearman approaches. The majority of key associations were consistent across all four methods. For example, the negative correlations between Actinobacteriota and salinity indicators (SAR, ESP, Na^+^) remained significant regardless of correlation method (all *p* < 0.05), as did the positive correlations between Actinobacteriota and nutrient availability (AN, AP). Similarly, Proteobacteria-AP positive correlations were robust across methods (Spearman r = −0.506, *p* = 0.007; Pearson r = −0.441, *p* = 0.021; Kendall τ = −0.368, *p* = 0.007; CLR-Spearman r = 0.162, *p* = 0.418). However, certain associations exhibited method-sensitivity. For instance, several Firmicutes-environment correlations that were significant under traditional methods became non-significant after CLR transformation suggesting potential influence of compositional effects. These method-sensitive associations are interpreted with caution in the Discussion. Full results are presented in Supplementary Table S7.

In summary, microbial community variation along the Luyang Lake gradient was associated with a suite of co-varying edaphic factors, including osmotic stress (EC, TDS), ionic toxicity (Na^+^, Cl^−^), sodicity (SAR, ESP), and nutrient depletion (OM, AN, AP, DOC). The strong correlations among these variables preclude attribution of community shifts to any single factor. Salinity-related variables were among the most strongly associated with community composition, but their effects are inherently confounded with nutrient limitation and other stressors in this observational design.

## 4. Discussion

### 4.1. Coupled Salinity–Ion Accumulation and Nutrient Depletion in an Inland Saline–Alkaline Wetland

As detailed in Section 3.1, the Luyang Lake gradient encompasses multiple co-varying edaphic stressors that characterize natural inland salinization. This section contextualizes these patterns within broader salinization frameworks, with attention to the constraints of observational designs for causal inference.

The Luyang Lake wetland represents an inland saline–alkaline system that differs from coastal wetlands affected by tidal inputs and from secondary salinized agricultural soils shaped by irrigation and fertilization history. In the present study, the three sampling groups formed a clear environmental gradient characterized by increasing EC and soluble ion concentrations, together with declining soil nutrient availability. Therefore, rather than interpreting the gradient as salinity alone, we describe it as a coupled salinity–ion and nutrient gradient within an alkaline soil background.

As detailed in Section 3.1, the gradient was characterized by distinct changes in sodicity, alkalinity, and carbon availability. These patterns indicate that the gradient represents a composite of multiple stressors rather than salinity alone. In low-salinity NT soils, relatively higher Ca availability is consistent with the reported role of Ca in maintaining soil aggregation and partially buffering sodium-induced structural changes in saline soils elsewhere [24]. In HB soils, the Ca^2+^/Na^+^ molar ratio increased from 0.49 in NT to 0.76 in HB, despite the rise in absolute Na^+^ concentrations. This pattern indicates that total Ca^2+^ availability was not the primary structural constraint; rather, the increase in exchangeable sodium (Ex-Na^+^: 1.35 to 5.09 cmol/kg) and ESP suggests that sodium dominance on soil colloid surfaces is associated with changes in soil aggregation. Elevated Mg^2+^ is associated with osmotic stress and reduced water availability for microorganisms under saline conditions [25]. However, because these stressors are inherently correlated in this natural gradient, their independent effects on microbial communities cannot be fully separated without manipulative experiments. For example, the decline in Glomeromycota could reflect osmotic stress, sodium toxicity, or reduced carbon availability, and the relative abundance increase in Gammaproteobacteria in HB is compatible with both salt tolerance and adaptation to low organic carbon, or both.

The reduced OM content in HB soils is consistent with several non-exclusive processes, including suppressed plant productivity, reduced litter input, slower microbial decomposition, and altered carbon stabilization under saline conditions [26,27]. The deterioration of soil structure and ion imbalance associated with high ESP is linked to constraints on microbial colonization and substrate diffusion [28,29]. Although pH remained alkaline across all samples (8.19–9.32), its variation was smaller than that of EC and soluble ions. This pattern is consistent with the observation that salinity-related variables showed stronger associations with microbial community composition than pH in this dataset [30]. However, this comparison is descriptive and does not establish that salinity is causally more important than pH.

Previous studies in arid and coastal wetlands have identified EC and major ions, especially Na^+^ and Cl^−^, as predictors of microbial community variation, often through osmotic stress and ionic toxicity [31,32]. Our results are consistent with these findings, but also indicate that microbial shifts in Luyang Lake occurred under simultaneous nutrient depletion. Unlike some coastal wetlands where tidal exchange can subsidize nutrients, the HB habitats in Luyang Lake showed both high salt accumulation and low carbon and nitrogen availability [33]. Thus, microbial responses in this inland wetland likely reflect the combined influence of salinity, ion composition, and nutrient limitation, rather than the effect of salinity alone. The observed pattern is consistent with a “salt accumulation–nutrient depletion” coupling, but the independent contribution of each factor requires further experimental validation [34,35].

### 4.2. Divergent Bacterial and Fungal Responses Along the Salinity Gradient

As detailed in Section 3.2 and Section 3.3, bacterial and fungal communities showed contrasting diversity patterns along the Luyang Lake gradient: bacteria exhibited a significant unimodal α-diversity response (quadratic regression: *p* < 0.001 for both Shannon and Chao1), whereas fungal Shannon diversity declined with increasing salinity and fungal Chao1 showed a U-shaped response (quadratic regression: Shannon β_2_ = −0.013, R^2^ = 0.28, *p* = 0.02; Chao1 β_2_ = +0.54, R^2^ = 0.71, *p* < 0.001). The discrepancy between fungal Shannon and Chao1 patterns highlights the context-dependency of diversity metrics: Shannon weights abundance evenness and is inherently dominated by common taxa, whereas Chao1 extrapolates total richness from singleton and doubleton frequencies and is thus disproportionately influenced by the rare biosphere. Because this environmental gradient coincides with marked vegetation turnover across sites (Table S1), these diversity patterns should be interpreted as salinity-associated rather than salinity-exclusive responses. The U-shaped Chao1 response in fungi—with elevated values at both salinity extremes and a minimum at intermediate salinity—suggests that extreme environments (both low-salinity NT and high-salinity HB) harbor reservoirs of rare fungal variants, possibly including dormant spores, stress-tolerant specialists, or transient colonizers from adjacent microhabitats. By contrast, intermediate salinity (ZZ) may represent a ‘mesic’ condition where competitive exclusion reduces the diversity of rare niches, lowering singleton/doubleton counts. This interpretation aligns with the ‘rare biosphere’ concept, which posits that extreme or fluctuating environments can maintain cryptic diversity through persistence of low-abundance, conditionally active taxa. Importantly, the robustness of our compositional findings was confirmed by applying centred log-ratio (clr) transformation and FDR-corrected Kruskal–Wallis tests, which address both the constant-sum constraint inherent to amplicon data and the multiple testing burden across taxa. Under this stringent framework, 30 of 47 bacterial phyla (63.8%) and 67 of 119 classes (56.3%) remained significantly differentially abundant among salinity groups (FDR-adjusted *p* < 0.05). This high proportion of significantly affected taxa underscores that salinity exerts pervasive selective pressure across diverse microbial lineages, rather than idiosyncratically affecting a few dominant taxa. The consistency of these results with our original relative abundance patterns suggests that the observed community shifts are biologically meaningful rather than artifacts of compositional bias.

The apparent contradiction between Ascomycota’s proportional dominance in HB and its absolute decline in relative abundance from NT to HB (Section 3.3) reflects a general challenge in interpreting relative abundance data in low-diversity communities. When total fungal ASV richness drops by approximately 80% (from 1282 in NT to 279 in HB), the few remaining taxa necessarily occupy a larger proportional share of the community, even if their absolute abundance or biomass has also declined. This “survivor dominance” effect has been reported in other saline ecosystems, where fungi often show stronger sensitivity to salinity than bacteria, and rare or stress-tolerant taxa may disproportionately contribute to community persistence [36]. However, this proportional dominance does not indicate functional compensation or ecosystem resilience; it simply describes the statistical consequence of diversity loss. We caution that statistically significant correlations in amplicon-derived networks may partly reflect technical artifacts—such as primer bias, variable amplification efficiency, or rarefaction noise—rather than true biological interactions or shared niches. While prevalence filtering, bootstrap validation, and rarefaction to even depth mitigate these issues, metagenomic or culture-based validation would be required to confirm that these patterns represent ecologically meaningful associations.

The stronger fungal sensitivity observed here is consistent with both microbial physiological differences and the concurrent turnover of host vegetation along the gradient. Fungal hyphal growth and spore germination can be vulnerable to osmotic stress, but fungal communities are also tightly linked to plant-derived carbon inputs, litter quality, and root-associated microsites [37]. Because the Luyang Lake gradient coincides with a transition from *Phragmites australis*-dominated vegetation to communities dominated by *Suaeda salsa* and *Kochia scoparia* (Table S1), the observed fungal decline could reflect a combination of direct edaphic stress and plant-mediated habitat restructuring. The present field survey cannot disentangle these pathways.

At the bacterial phylum level, the observed shifts—Proteobacteria and Actinobacteriota showing significant opposite trends (both FDR-adjusted *p* < 0.003 after clr-transformation)—are descriptive of community reorganization along the gradient. Notably, our compositional analysis revealed that even rare phyla, including Entotheonellaeota (FDR-adjusted *p* < 0.001), Spirochaetota (FDR-adjusted *p* < 0.001), and Abditibacteriota (FDR-adjusted *p* = 0.003), showed highly significant responses to salinity, indicating that environmental filtering operates across the full phylogenetic breadth of the bacterial community rather than selectively targeting dominant groups. Some studies have linked these phyla to saline or nutrient-poor conditions, but phylum-level taxonomy is too coarse to infer physiological mechanisms. For example, Proteobacteria encompasses metabolically diverse lineages ranging from oligotrophic Alphaproteobacteria to copiotrophic Gammaproteobacteria, and the observed increase in HB may reflect different ecological strategies within this phylum. Similarly, Chloroflexi and Gemmatimonadota have been reported from arid and saline soils [38,39], but their presence does not demonstrate adaptation to ion stress; it is equally compatible with tolerance to low organic carbon or other correlated factors. We therefore caution against interpreting phylum-level abundance shifts as evidence of uniform physiological strategies.

The decline of Mortierellomycota and Glomeromycota in HB soils is consistent with their association with nutrient-rich soils and active plant–microbe interactions, respectively. However, because the salinity gradient in this study is accompanied by substantial turnover in above-ground vegetation (Table S1), these fungal shifts may reflect not only edaphic stress but also changes in litter inputs, root architecture, and rhizosphere habitat quality. The decrease in Glomeromycota is compatible with altered plant cover and root-associated environments, but vegetation composition, root biomass, and root exudation were not quantified in this study. Therefore, these patterns should be interpreted as salinity-associated and vegetation-coupled changes rather than direct evidence of salinity alone weakening plant-fungal symbiosis.

The observation that bacteria maintained greater compositional flexibility—with over 60% of phyla responding significantly to salinity even after conservative FDR correction—while fungi showed stronger richness loss agrees with studies from other saline regions. However, this domain-level contrast may be context-dependent. Bacterial metabolic diversity is broad, but individual bacterial lineages can be as sensitive to salinity as fungi; the apparent “flexibility” may simply reflect higher bacterial species richness and functional redundancy, which buffer against diversity loss. Conversely, fungal sensitivity may be exaggerated by the ITS-based analysis, which has lower phylogenetic resolution than bacterial 16S and may miss cryptic fungal diversity or fail to distinguish dead from active biomass.

Differences from coastal wetlands, such as the Yellow River Delta, may arise from contrasting hydrological connectivity, dispersal sources, and nutrient inputs [40]. In coastal systems, tidal exchange can introduce salt-tolerant microbial taxa from marine sources and subsidize nutrients, potentially sustaining higher fungal diversity than observed in the isolated, nutrient-depleted Luyang Lake system. This comparison highlights that “salinity sensitivity” is not an intrinsic property of fungal communities but depends on the biogeochemical context in which salinity operates.

### 4.3. Assembly Processes and Co-Occurrence Patterns Under High Salinity

Community assembly analyses indicated that bacterial communities showed stronger deterministic signals in HB than in NT or ZZ. The Neutral Community Model showed that stochastic processes contributed substantially to bacterial community structure, especially under NT and ZZ conditions. iCAMP analysis indicated that deterministic processes (homogeneous and heterogeneous selection) were more prevalent in HB soils compared to NT and ZZ. This pattern is consistent with studies in saline agricultural soils and arid ecosystems reporting similar transitions [41]. However, several caveats limit interpretation. First, iCAMP quantifies assembly processes at the phylogenetic-bin level; the higher prevalence of homogeneous selection in HB reflects bin-level phylogenetic clustering, not species-specific responses. Because bins may contain multiple taxa with diverse ecological strategies, attributing this pattern to specific lineages or traits is not supported by the analysis. Second, stochastic processes remained substantial in HB (approximately 51% for bacteria), indicating that environmental filtering, while more prevalent, did not exclusively determine community composition. Third, the gradient design cannot distinguish whether the higher prevalence of deterministic processes reflects a linear response to increasing salinity or a threshold effect at which only tolerant taxa persist.

The contraction of bacterial niche breadth in HB soils (Section 3.4) is compatible with the expectation that high salinity is associated with a reduced range of viable ecological strategies. However, niche breadth is calculated from ASV occurrence patterns across samples, not from direct measurements of physiological tolerance. A decline in niche breadth may therefore reflect reduced habitat heterogeneity among HB sites (all were highly saline), rather than intrinsic physiological constraints on individual taxa. This distinction is important: if HB sites were more environmentally homogeneous than NT or ZZ sites, the observed niche breadth contraction may be a sampling artifact rather than an ecological response.

The inferred physiological mechanisms—osmotic adjustment, ion homeostasis, and efficient substrate use—are compatible with the taxonomic patterns observed in HB, but they remain speculative [42]. Functional genes and physiological traits were not directly measured, and the presence of salt-tolerant taxa does not demonstrate that these mechanisms are active. Future metagenomic or metatranscriptomic analyses would be needed to verify whether osmoprotection, ion transport, or carbon acquisition pathways are enriched in HB communities.

Building on the descriptive network patterns reported in Section 3.5, we emphasize that the following interpretation focuses on inferred statistical associations rather than verified ecological interactions or ecosystem functions. Co-occurrence network analysis showed that microbial association patterns were less complex under high salinity, with fewer nodes, edges, and highly connected taxa in HB compared to NT and ZZ. In ZZ soils, negative cohesion was higher than in NT or HB, a pattern that is statistically consistent with reduced shared environmental preferences or increased niche differentiation, but that is equally compatible with independent responses to the gradient or compositional effects in relative abundance data [43]. The stress-gradient hypothesis, which predicts stronger negative interactions under moderate stress, is one possible interpretation, but correlation-based networks cannot distinguish competition from habitat filtering or random co-occurrence.

In HB soils, both positive and negative cohesion approached low levels, consistent with sparse correlation structure among the few remaining taxa. This reduction may reflect biological factors (reduced diversity, environmental filtering), methodological factors (increased data sparsity, threshold effects, and mathematical constraints on correlation detection in low-richness assemblages), or both. The networks were inferred from Spearman correlations and therefore represent statistical co-occurrence rather than direct biological interactions. Shared responses to environmental gradients, unmeasured vegetation effects, and compositional data constraints may all influence correlation-based networks. Cohesion summarizes the strength of associations in the inferred network, but it does not measure facilitation, competition, or ecosystem stability. The observed reduction in network complexity should therefore be described as a simplification of inferred microbial association structure.

Despite these limitations, the network results are broadly consistent with the diversity and assembly analyses. High salinity was associated with reduced microbial richness and fewer inferred statistical associations. The observed network simplification in HB may arise from multiple non-exclusive factors: (i) reduced ASV richness inherently limits the number of possible pairwise correlations (mathematical constraint); (ii) increased data sparsity in low-diversity communities reduces correlation detection power (methodological constraint); and (iii) environmental filtering may reduce shared habitat preferences among the few remaining taxa (biological hypothesis). Because these factors are confounded in this observational design, the relative contribution of each cannot be determined. We therefore describe the network patterns as statistical association structures rather than as evidence of altered ecological interactions or ecosystem function. This differs from rhizosphere systems, where root exudates can support dense microbial networks. However, this comparison is speculative: root biomass, exudation, and rhizosphere chemistry were not measured in this study, and the observed network simplification may reflect data sparsity as much as any biological effect.

### 4.4. Implications for Wetland Restoration and Future Research

Several limitations should be acknowledged. First, although SAR, ESP, carbonate/bicarbonate chemistry, soil water content, DOC, and inorganic N were measured (Table 1), these variables are strongly correlated with EC and each other along the natural gradient. This multicollinearity prevents rigorous attribution of microbial responses to individual stressors (osmotic vs. sodicity vs. alkalinity vs. nutrient limitation) without factorial experiments or path analysis. Second, although aboveground vegetation metrics were comparable among groups (Table S1), root biomass, root exudation, and rhizosphere chemistry were not quantified. Because dominant plant species shifted from *Phragmites australis*/*Cynodon dactylon* to *Suaeda glauca*/*Kochia scoparia*, species-specific root inputs may have confounded edaphic effects. Third, and most critically for generalizability, sampling was conducted in a single season (October 2023). Inland wetland salinity, soil moisture, and microbial metabolic activity exhibit strong seasonal fluctuations in semi-arid climates, particularly during spring snowmelt and summer evaporation peaks. The observed patterns therefore represent a static snapshot that may not reflect year-round community dynamics. This temporal limitation is a major constraint on the inference of stable salinity–microbiome relationships and underscores the need for multi-seasonal monitoring to validate the patterns reported here. Fourth, the ITS-based fungal phylogeny used in iCAMP is less conserved than bacterial 16S rRNA; fungal assembly results should therefore be interpreted cautiously [44].

The observed microbial patterns along the Luyang Lake gradient are consistent with a “salt accumulation–nutrient depletion” coupling that may constrain restoration outcomes, but these implications are hypothetical rather than empirically tested. Hydrological management to reduce surface salt accumulation, organic carbon and nutrient inputs to alleviate substrate limitation, and vegetation reconstruction to restore root-associated carbon flows are all plausible restoration strategies. However, none were evaluated in this study, and their effectiveness would depend on site-specific factors including drainage efficiency, organic matter quality, and plant–microbe compatibility. We therefore present these as hypotheses for future restoration research rather than as recommendations for management action.

The shift in dominant vegetation from *P. australis*/*C. dactylon* to *S. glauca*/*K. scoparia* along the gradient raises additional questions for restoration. Aboveground vegetation metrics did not differ significantly among groups (Table S1), but species-specific root traits, exudation chemistry, and rhizosphere effects were not measured. Future studies incorporating root biomass, root exudates, and rhizosphere metabolites would be needed to disentangle plant-mediated and edaphic effects on microbial communities.

We acknowledge that the salinity gradient in our study is concomitant with a complete turnover of the above-ground plant community, from *Phragmites australis*-dominated stands at low salinity to *Suaeda salsa-* and *Kochia scoparia*-dominated stands at higher salinity (Table S1). Consequently, the observed microbial shifts cannot be unambiguously attributed to edaphic salinity alone. Plant-mediated habitat filtering, including altered root exudate composition, litter chemistry, rhizosphere oxygen conditions, and root-derived carbon inputs, likely co-determines microbial diversity and community composition along this gradient. Disentangling the relative contributions of salinity and vegetation turnover would require factorial microcosm experiments, vegetation-controlled transplant designs, or paired rhizosphere-bulk soil sampling beyond the scope of the present field survey.

This study highlights several priorities for future work. First, factorial experiments that independently manipulate salinity, nutrient availability, and plant identity would help resolve whether microbial shifts are driven by osmotic stress, ionic toxicity, carbon limitation, vegetation turnover, or their interactions. Second, temporal sampling across seasons would capture whether the observed patterns are stable or transient. Third, metagenomic or metatranscriptomic analyses would move beyond taxonomic patterns to identify functional pattern of salt tolerance and nutrient acquisition. Fourth, comparative studies across inland and coastal saline wetlands would test whether the “salt accumulation–nutrient depletion” coupling observed here is a general feature of inland systems or specific to the Luyang Lake context.

## 5. Conclusions

Primary salinization in Luyang Lake is associated with substantial changes in soil microbial community structure along the measured gradient. The transition from relatively diverse, stochastically assembled, and densely interconnected communities in low-to-moderate-salinity soils to low-diversity, sparsely associated assemblages with stronger deterministic signals in hypersaline soils describes a shift in community organization that is consistent with stronger environmental filtering under high salinity. These patterns are descriptive of the sampled gradient and do not demonstrate loss of ecosystem function, resilience, or recovery capacity. The co-occurrence of high salinity and severe nutrient depletion in hypersaline soils raises questions about the reversibility of observed microbial shifts. Whether the simplified community structures in HB represent a stable alternative state or a transient response to chronic stress remains unresolved. The relative importance of osmotic stress, ionic toxicity, and carbon limitation in constraining microbial recovery cannot be determined from this observational design. From a restoration perspective, the “salt accumulation–nutrient depletion” coupling identified in this study suggests that interventions focusing on salt leaching alone may be insufficient if nutrient availability remains limiting. However, this inference is hypothetical: the effectiveness of combined salt and nutrient management in promoting microbial community recovery has not been tested in this system. Factorial experiments that independently manipulate salinity and nutrient availability, coupled with long-term monitoring of community composition and functional activity, would be needed to evaluate whether the observed patterns are reversible and whether restoration outcomes can be improved by addressing multiple stressors simultaneously.

## Figures and Tables

**Table 1:** Soil physical and chemical properties of Luyang Lake Wetland.

Salinity-Alkalinity Levels	NT (Non-Saline)	ZZ (Moderately Saline)	HB (Hypersaline Soils)
pH	8.61 ± 0.32	8.89 ± 0.16	9.04 ± 0.18
Soil water content (%)	17.05 ± 0.50	18.79 ± 0.72	20.35 ± 0.94
EC (ms/cm)	0.40 ± 0.18	2.95 ± 1.20	7.62 ± 1.46
TDS (mg/kg)	253.87 ± 114.16	1886.72 ± 764.82	4874.67 ± 935.25
Osmotic potential (MPa)	−0.01 ± 0.01	−0.11 ± 0.04	−0.27 ± 0.05
SO_4_^2−^(cmol/kg)	0.32 ± 0.19	7.13 ± 2.46	17.24 ± 3.13
Ca^2+^ (cmol/kg)	0.86 ± 0.21	2.89 ± 1.35	5.85 ± 0.83
Mg^2+^ (cmol/kg)	3.26 ± 0.75	5.15 ± 0.45	6.63 ± 0.72
Na^+^ (cmol/kg)	1.75 ± 0.42	4.09 ± 0.47	7.73 ± 1.08
Cl^−^ (cmol/kg)	0.31 ± 0.23	6.05 ± 2.50	24.28 ± 8.99
SAR	0.83 ± 0.24	2.06 ± 0.29	4.00 ± 0.56
ESP (%)	10.39 ± 1.48	11.20 ± 0.64	18.00 ± 2.22
Total alkalinity (mg/kg)	236.25 ± 14.07	320.11 ± 16.07	417.54 ± 15.45
Exch. Na (cmol/kg)	1.35 ± 0.08	2.41 ± 0.09	5.09 ± 0.49
CEC (cmol/kg)	13.12 ± 1.21	21.57 ± 0.90	28.38 ± 1.18
Base saturation (%)	83.59 ± 14.05	56.43 ± 8.32	53.65 ± 4.42
CO_3_^2−^ (mg/kg)	1.73 ± 1.02	40.18 ± 3.06	46.34 ± 5.32
HCO_3_^−^ (mg/kg)	234.52 ± 14.30	279.92 ± 14.68	371.19 ± 10.90
OM (g/kg)	20.13 ± 5.11	9.16 ± 1.84	5.57 ± 0.59
DOC (mg/kg)	250.76 ± 83.11	74.26 ± 19.84	54.79 ± 14.49
SOC (g/kg)	11.68 ± 2.96	5.31 ± 1.07	3.23 ± 0.34
AP (mg/kg)	40.21 ± 6.88	24.41 ± 1.71	13.47 ± 1.91
AN (mg/kg)	93.20 ± 21.30	43.81 ± 4.84	18.97 ± 7.96
AK (mg/kg)	290.89 ± 39.34	185.89 ± 39.09	164.89 ± 24.13
Inorganic N (mg/kg)	25.47 ± 1.06	24.03 ± 0.66	21.93 ± 1.18

## Data Availability

The original contributions presented in this study are included in the article and Supplementary Material. Further inquiries can be directed to the corresponding author.

## Supplementary Materials

The following supporting information can be downloaded at https://www.mdpi.com/article/10.3390/microorganisms14071602/s1. Figure S1: Quadratic regression of bacterial (A, B) and fungal (C, D) α-diversity indices against electrical conductivity (EC). Figure S2: Within-site clustering confirms non-independence among quadrats. Figure S3: Intraclass correlation coefficients (ICC) quantify site-level variance across edaphic and biological variables. Figure S4: Consistency check between LMM and site-level ANOVA. Figure S5: Conservative denominator degrees of freedom (DenDF) in LMM versus naive ANOVA. Figure S6: LMM emmeans versus site-level means for Bac_Shannon. Figure S7: Significantly differentially abundant bacterial classes along the salinity gradient. Figure S8: Statistical analysis of fungal community dynamics. Figure S9: iCAMP analysis of community assembly processes at the phylogenetic-bin level (ds = 0.2). Figure S10: Topological evolution and destabilization of Fungi co-occurrence networks under saline-alkali stress. Table S1: Vegetation characteristics across salinity gradients in the Luyang Lake wetland. Table S2: Soil physical and chemical properties of Luyang Lake Wetland; Table S3: Bacterial network node attributes; Table S4: Fungal network node attributes; Table S5: Statistical results of phylum-level differential abundance analysis using clr-transformed data with FDR correction; Table S6: Statistical results of class-level differential abundance analysis using clr-transformed data with FDR correction. Table S7: Taxa environment sensitivity analysis.

## References

[1] Zhao Z., Song T., Zhang M., Tong S., An Y., Zhang P., Sang B., Cao G. (2023). Benefits of Morphology-Based Functional Group Classification to Study Dynamic Changes in Phytoplankton in Saline-Alkali Wetlands, Taking Typical Saline-Alkali Wetlands in Northeast China as an Example. Diversity.

[2] Chen H., Meng F., Sa C., Luo M., Zhang H., Bao S., Liu G., Bao Y. (2023). Synergistic Change and Driving Mechanisms of Hydrological Processes and Ecosystem Quality in a Typical Arid and Semi-Arid Inland River Basin, China. Remote Sens..

[3] Fang S., Hou X., Liang X. (2021). Response Mechanisms of Plants Under Saline-Alkali Stress. Front. Plant Sci..

[4] Guo Y., Dong J., Wang J., Meng Q., Ma J., Luqmon S., Gong P., Reaihan E., Liu H. (2025). Regional adaptability and integrated evaluation of saline-alkali soil remediation technologies: A comprehensive review. Ecol. Indic..

[5] Romero F., Labouyrie M., Orgiazzi A., Ballabio C., Panagos P., Jones A., Tedersoo L., Bahram M., Eisenhauer N., Sünnemann M. (2025). The soil microbiome as an indicator of ecosystem multifunctionality in European soils. Nat. Commun..

[6] Feng W., Yang J., Xu L., Zhang G. (2024). The spatial variations and driving factors of C, N, P stoichiometric characteristics of plant and soil in the terrestrial ecosystem. Sci. Total Environ..

[7] Stegen J., Lin X., Konopka A., Fredrickson J. (2012). Stochastic and deterministic assembly processes in subsurface microbial communities. ISME J..

[8] Huber P., Metz S., Unrein F., Mayora G., Sarmento H., Devercelli M. (2020). Environmental heterogeneity determines the ecological processes that govern bacterial metacommunity assembly in a floodplain river system. ISME J..

[9] Jewell M., Bell G. (2022). A basic community dynamics experiment: Disentangling deterministic and stochastic processes in structuring ecological communities. Ecol. Evol..

[10] Zhao S., Liu J., Banerjee S., Zhou N., Zhao Z., Zhang K., Tian C. (2018). Soil pH is equally important as salinity in shaping bacterial communities in saline soils under halophytic vegetation. Sci. Rep..

[11] Leonov V. (2023). Stochastic and Deterministic Processes in the Establishment of Taxonomic, Functional and Phylogenetic Diversity of Ecological Communities: A Review of Modern Concepts. Russ. J. Ecol..

[12] Ma B., Wang Y., Ye S., Liu S., Stirling E., Gilbert J., Faust K., Knight R., Jansson J., Cardona C. (2020). Earth microbial co-occurrence network reveals interconnection pattern across microbiomes. Microbiome.

[13] Hernandez D., David A., Menges E., Searcy C., Afkhami M. (2021). Environmental stress destabilizes microbial networks. ISME J..

[14] Arif Y., Singh P., Siddiqui H., Bajguz A., Hayat S. (2020). Salinity induced physiological and biochemical changes in plants: An omic approach towards salt stress tolerance. Plant Physiol. Biochem..

[15] Amit G., Bashan A. (2023). Top-down identification of keystone taxa in the microbiome. Nat. Commun..

[16] Banerjee S., Schlaeppi K., van der Heijden M. (2018). Keystone taxa as drivers of microbiome structure and functioning. Nat. Rev. Microbiol..

[17] Yan Y., Zhou J., He Z., Sun Q., Fei J., Zhou X., Zhao K., Yang L., Long H., Zheng H. (2017). Evolution of Luyang Lake since the last 34,000 years: Climatic changes and anthropogenic impacts. Quat. Int..

[18] Cheng Z., Shi J., He Y., Wu L., Xu J. (2022). Assembly of root-associated bacterial community in cadmium contaminated soil following five-year consecutive application of soil amendments: Evidences for improved soil health. J. Hazard Mater..

[19] Cheng Z., Shi J., He Y., Chen Y., Wang Y., Yang X., Wang T., Wu L., Xu J. (2023). Enhanced soil function and health by soybean root microbial communities during in situ remediation of Cd-contaminated soil with the application of soil amendments. mSystems.

[20] Chen W., Ren K., Isabwe A., Chen H., Liu M., Yang J. (2019). Stochastic processes shape microeukaryotic community assembly in a subtropical river across wet and dry seasons. Microbiome.

[21] Ning D., Yuan M., Wu L., Zhang Y., Guo X., Zhou X., Yang Y., Arkin A., Firestone M., Zhou J. (2020). A quantitative framework reveals ecological drivers of grassland microbial community assembly in response to warming. Nat. Commun..

[22] Fan Q., Liu K., Wang Z., Liu D., Li T., Hou H., Zhang Z., Chen D., Zhang S., Yu A. (2024). Soil microbial subcommunity assembly mechanisms are highly variable and intimately linked to their ecological and functional traits. Mol. Ecol..

[23] Sun W., Jing Z. (2023). Migration of rare and abundant species, assembly mechanisms, and ecological networks of microbiomes in drinking water treatment plants: Effects of different treatment processes. J. Hazard. Mater..

[24] Eissa M., Alotaibi M., Alotibi M., Aljuaid A., Aldayel T., Ghoneim A. (2025). A Novel Biostimulant-Biochar Strategy for Improving Soil Quality and Salinity Tolerance in Medicinal Mint (*Mentha longifolia* L.). Soil Syst..

[25] Choudhary S., Kumar V., Singhal R., Bose B., Chauhan J., Alamri S., Siddiqui M., Javed T., Shabbir R., Rajendran K. (2021). Seed Priming with Mg(NO_3_)_2_ and ZnSO_4_ Salts Triggers the Germination and Growth Attributes Synergistically in Wheat Varieties. Agronomy.

[26] Shu W., Huang L. (2022). Microbial diversity in extreme environments. Nat. Rev. Microbiol..

[27] Morrissey E., Gillespie J., Morina J., Franklin R. (2014). Salinity affects microbial activity and soil organic matter content in tidal wetlands. Glob. Change Biol..

[28] Guo H., Huang Z., Li M., Hou Z. (2020). Growth, ionic homeostasis, and physiological responses of cotton under different salt and alkali stresses. Sci. Rep..

[29] Xie Y., Ning H., Zhang X., Zhou W., Xu P., Song Y., Li N., Wang X., Liu H. (2024). Reducing the Sodium Adsorption Ratio Improves the Soil Aggregates and Organic Matter in Brackish-Water-Irrigated Cotton Fields. Agronomy.

[30] Gupta B., Huang B. (2014). Mechanism of Salinity Tolerance in Plants: Physiological, Biochemical, and Molecular Characterization. Int. J. Genom..

[31] Zhao X., Gao J., Yu X., Borjigin Q., Qu J., Zhang B., Zhang S., Li Q., Guo J., Li D. (2024). Evaluation of the microbial community in various saline alkaline-soils driven by soil factors of the Hetao Plain, Inner Mongolia. Sci. Rep..

[32] Zhang X., Zhai H., Xu H., Song W., Liu M., Dong X., Sun W., Ma J. (2025). Different salt stress types regulated rhizosphere rare bacterial communities through root exudates and soil physicochemical properties. Plant Soil.

[33] Yang H., Jing M., Sheng H., Liu M., Yang W., Lu K., Zhu J. (2025). Temporal niche differentiation of rhythmic taxa enhances microbial network stability in a subtropical reservoir. Environ. Res..

[34] Shabtai I., Wilhelm R., Schweizer S., Höschen C., Buckley D., Lehmann J. (2023). Calcium promotes persistent soil organic matter by altering microbial transformation of plant litter. Nat. Commun..

[35] Marschner P. (2021). Processes in submerged soils—Linking redox potential, soil organic matter turnover and plants to nutrient cycling. Plant Soil.

[36] Rao M., Luo Z., Dong Z., Li Q., Liu B., Guo S., Nie G., Li W. (2022). Metagenomic analysis further extends the role of Chloroflexi in fundamental biogeochemical cycles. Environ. Res..

[37] He Y., Liu C., Ni B., Lian H. (2024). Ecological Network Resilience of Shiyang River Basin: An Arid Inland Watershed of Northwest China. Chin. Geogr. Sci..

[38] Papanikolopoulou L., Smeti E., Roelke D., Dimitrakopoulos P., Kokkoris G., Danielidis D., Spatharis S. (2018). Interplay between r- and K-strategists leads to phytoplankton underyielding under pulsed resource supply. Oecologia.

[39] Ma Y., Zhao H., Shan Q., Xu Y., Yu M., Cui J., Liu T., Qiao L., He X. (2021). K-strategy species plays a pivotal role in the natural attenuation of petroleum hydrocarbon pollution in aquifers. J. Hazard. Mater..

[40] Wei Y., Chen L., Yin Z., Feng Q., Xi H., Zhang C., Gan K., Yong T. (2024). Differences in soil fungal communities under salinity gradients in arid and semiarid regions. Glob. Planet. Change.

[41] Li P., Xu T., Hu Q., Gu S., Yang Y., Wang Z., Deng X., Wang B., Li W., Zhu Y. (2023). Spatial distribution pattern across multiple microbial groups along an environmental stress gradient in tobacco soil. Ann. Microbiol..

[42] Gao G., Li G., Liu M., Li P., Liu J., Ma S., Li D., Petropoulos E., Wu M., Li Z. (2023). Changes in soil stoichiometry, soil organic carbon mineralization and bacterial community assembly processes across soil profiles. Sci. Total Environ..

[43] Yang C., Sun J. (2020). Soil Salinity Drives the Distribution Patterns and Ecological Functions of Fungi in Saline-Alkali Land in the Yellow River Delta, China. Front. Microbiol..

[44] Jiang Z., Zhang P., Wu Y., Wu X., Ni H., Lu Q., Zang S. (2024). Long-term surface composts application enhances saline-alkali soil carbon sequestration and increases bacterial community stability and complexity. Environ. Res..

