# Supplementary material for: Patterns of Soil Microbial Diversity, Assembly, and Co-Occurrence Along a Natural Salinity Gradient in an Inland Saline–Alkali Wetland
Source: Microorganisms. 2026 Jul 22;14(7):1602. doi: 10.3390/microorganisms14071602 (PMC13414336; doi:10.3390/microorganisms14071602)
Supplement: Supplementary file 1 [file microorganisms-14-01602-s001.zip › Supplementary Materials-revise3 .pdf]

Supplementary Information for

Patterns of Soil Microbial Diversity, Assembly, and Co-Occurrence Along a Natural Salinity Gradient in an Inland Saline–Alkali Wetland

Wei Jie[1]*, Chang Fan[2], Yang Haomin[3], Sun Yan[4], Li Zhi[4], Li Jun[1], Liu Nannan[5], Hao Zhuan[1]

[1]College of Environmental and Life Sciences, Weinan Normal University, Weinan 714009, China;

[2]Shaanxi Institute of Microbiology, Xi'an, 710043, China.

[3]Fisheries Institute, Sichuan Academy of Agricultural Sciences, Yibin, 644000, China

[4]College of Life Sciences, Shaanxi Normal University, Xi'an, 710119, PR China.

[5]Key Laboratory for Ecology and Environment of River Wetlands in Shaanxi Province, Weinan

*** Corresponding author:**

Wei Jie, School of Environmental and Life Sciences, Weinan Normal University, Weinan 714009, China. E-mail: weijie@snnu.edu.cn. https://orcid.org/0000-0001-5963-0339

Supplementary Tables

Table S1: Vegetation characteristics across salinity gradients in the Luyang Lake wetland.

Table S2: Soil physical and chemical properties of Luyang Lake Wetland;

Table S3: Bacterial network node attributes;

Table S4: Fungal network node attributes;

Table S5: Statistical results of phylum-level differential abundance analysis using clr-transformed data with FDR correction;

Table S6: Sta-tistical results of class-level differential abundance analysis using clr-transformed data with FDR correction.

Table S7: Taxa environment sensitivity analysis.

Supplementary Figures:

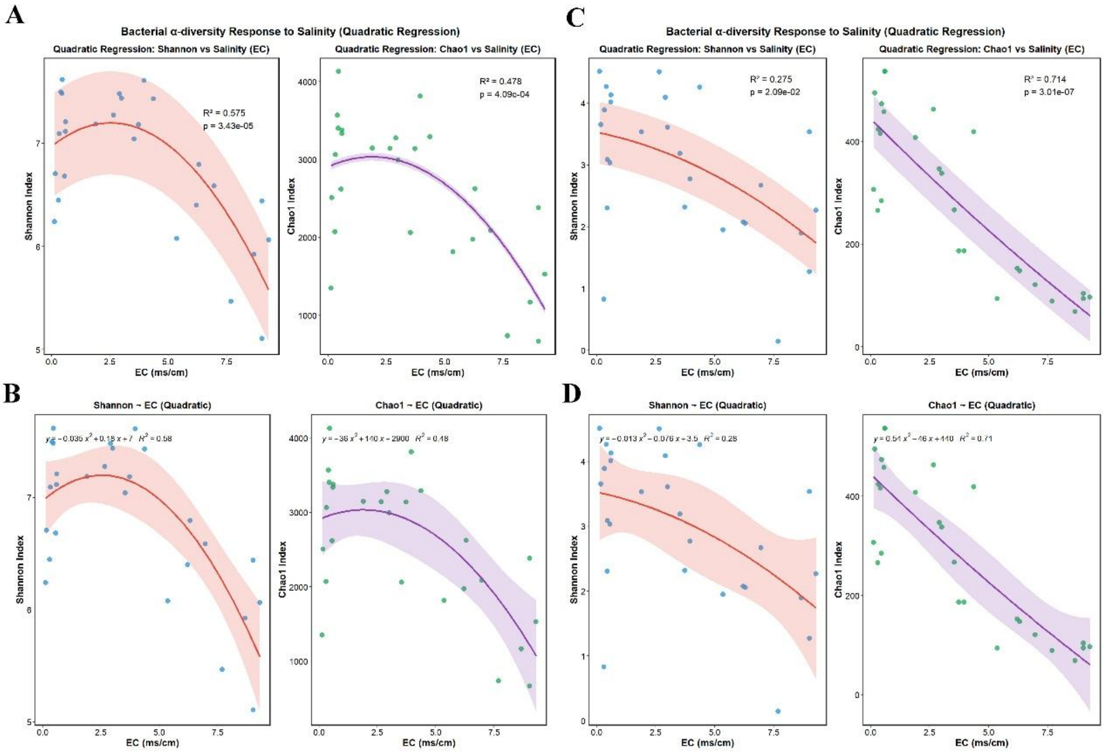

Figure S1. Quadratic regression of bacterial (A, B) and fungal (C, D) α-diversity indices against electrical conductivity (EC). A, C Regression on site-level means (n = 9) with model R^2 and overall P values. B, D Equivalent plots with regression equations displayed. Bacterial Shannon and Chao1 showed significant unimodal responses ($\beta_2 < 0$, $P < 0.001$). Fungal Shannon showed a weak quadratic fit consistent with monotonic decline ($\beta_2 = -0.013$, $R^2 = 0.28$, $P = 0.02$), whereas fungal

Chao1 exhibited a U-shaped response ($\beta_2 = +0.54$, $R^2 = 0.71$, $P < 0.001$).

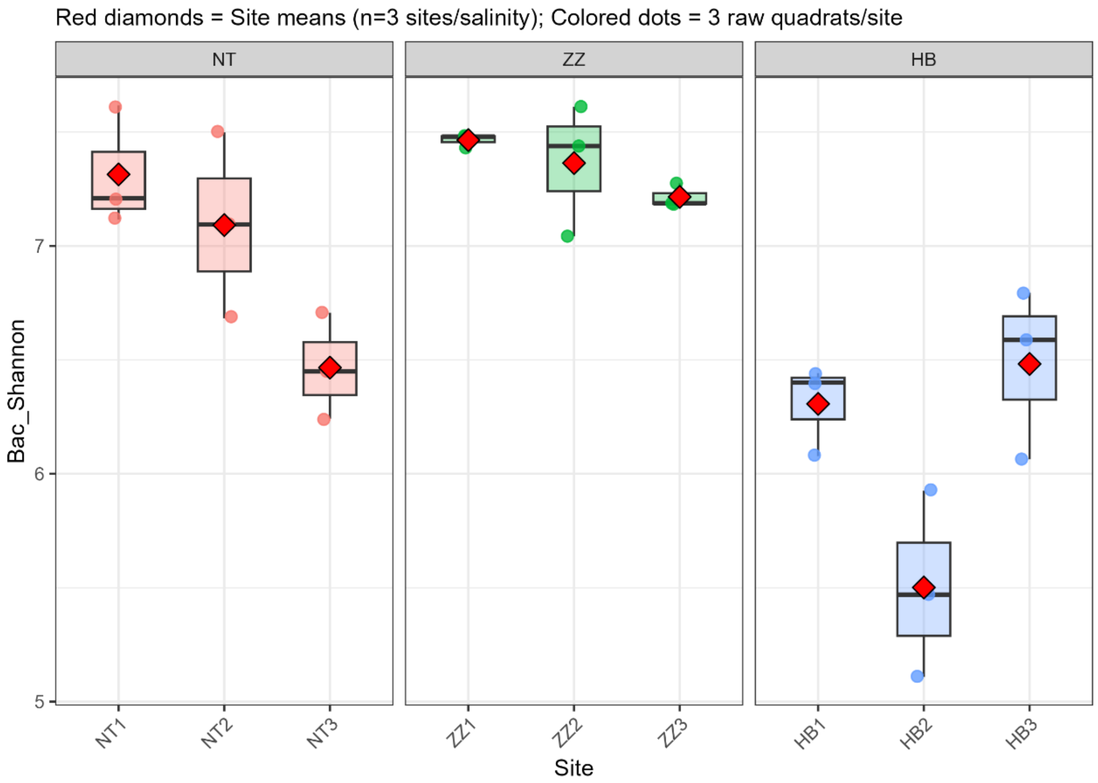

Figure S2. Within-site clustering confirms non-independence among quadrats. Boxplots show bacterial Shannon diversity (Bac_Shannon) for each site (NT1–NT3, ZZ1–ZZ3, HB1–HB3) grouped by salinity level (NT = low, ZZ = moderate, HB = high). Red diamonds denote site means (n = 3 sites per salinity); colored circles represent the three raw quadrats per site. The tight clustering of quadrats around their respective site means visually confirms that subsamples within sites are not statistically independent, supporting the inclusion of Site as a random factor.

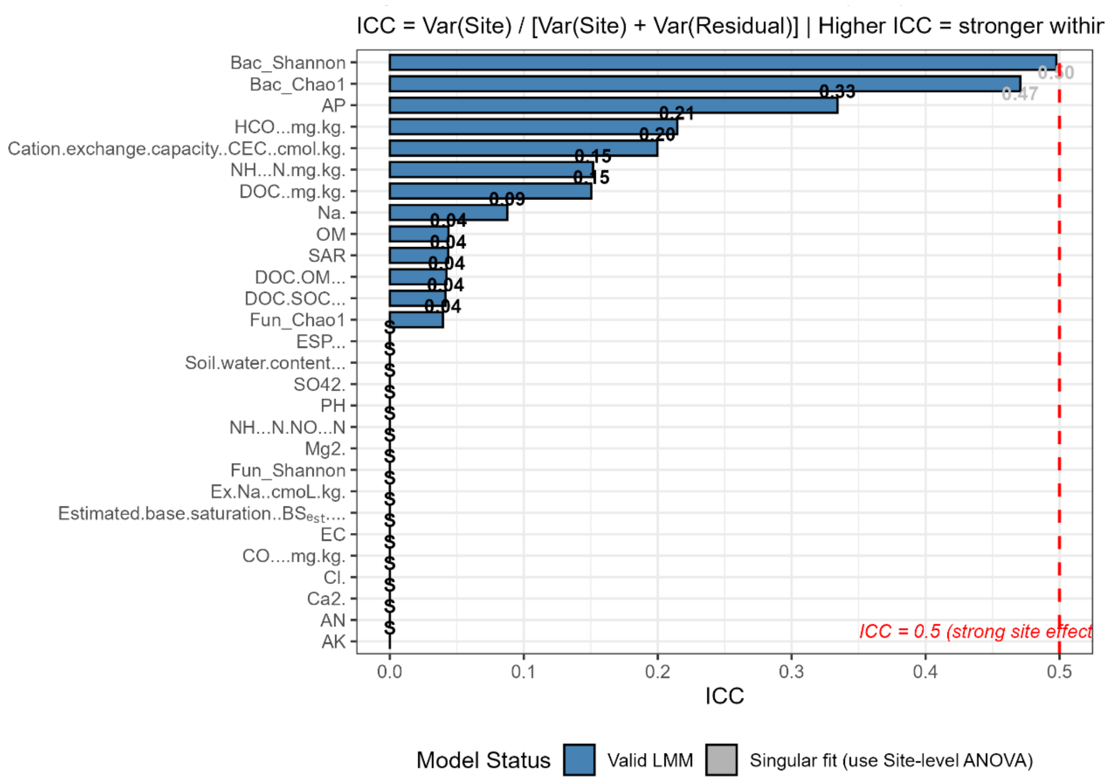

Figure S3. Intraclass correlation coefficients (ICC) quantify site-level variance across edaphic and biological variables. ICC = Var(Site) / [Var(Site) + Var(Residual)]; higher values indicate stronger within-site clustering and greater risk of pseudoreplication if Site is ignored. Blue bars denote valid linear mixed-effects models (LMM); grey bars denote singular fits for which site-level ANOVA was substituted. Bacterial diversity indices exhibited the strongest site effects (Bac_Shannon ICC = 0.50; Bac_Chao1 ICC = 0.47), approaching or exceeding the conventional threshold for strong site dependence (ICC = 0.5, red dashed line).

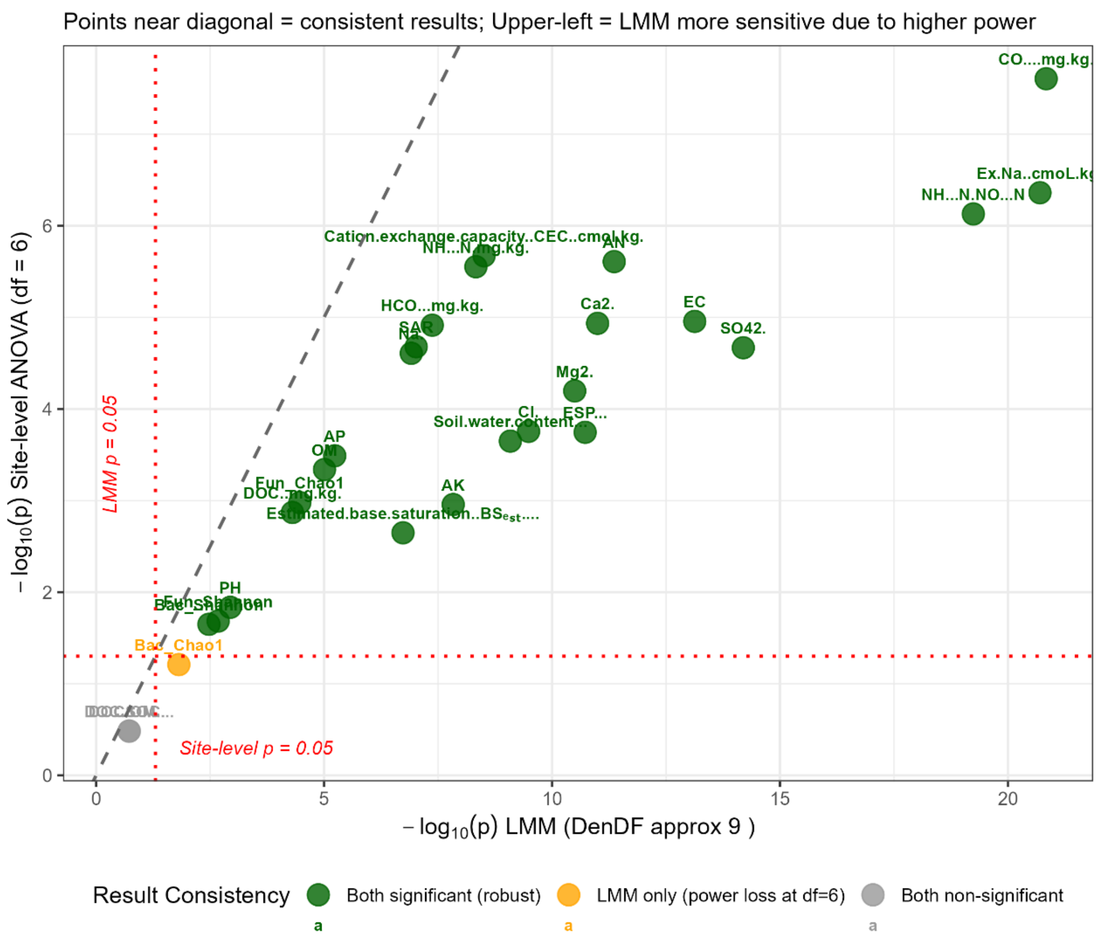

Figure S4. Consistency check between LMM and site-level ANOVA. Each point represents one response variable. The x-axis shows $-\log_{10}(p)$ from LMM with Satterthwaite approximation (DenDF $\approx$ 9); the y-axis shows $-\log_{10}(p)$ from site-level ANOVA (df = 6). The dashed diagonal marks equivalence. Green circles: significant in both tests (robust results); orange circles: significant only in LMM (power gain without Type I error inflation); grey circles: non-significant in both. The majority of points fall near the diagonal, confirming that LMM p-values are not inflated by pseudoreplication.

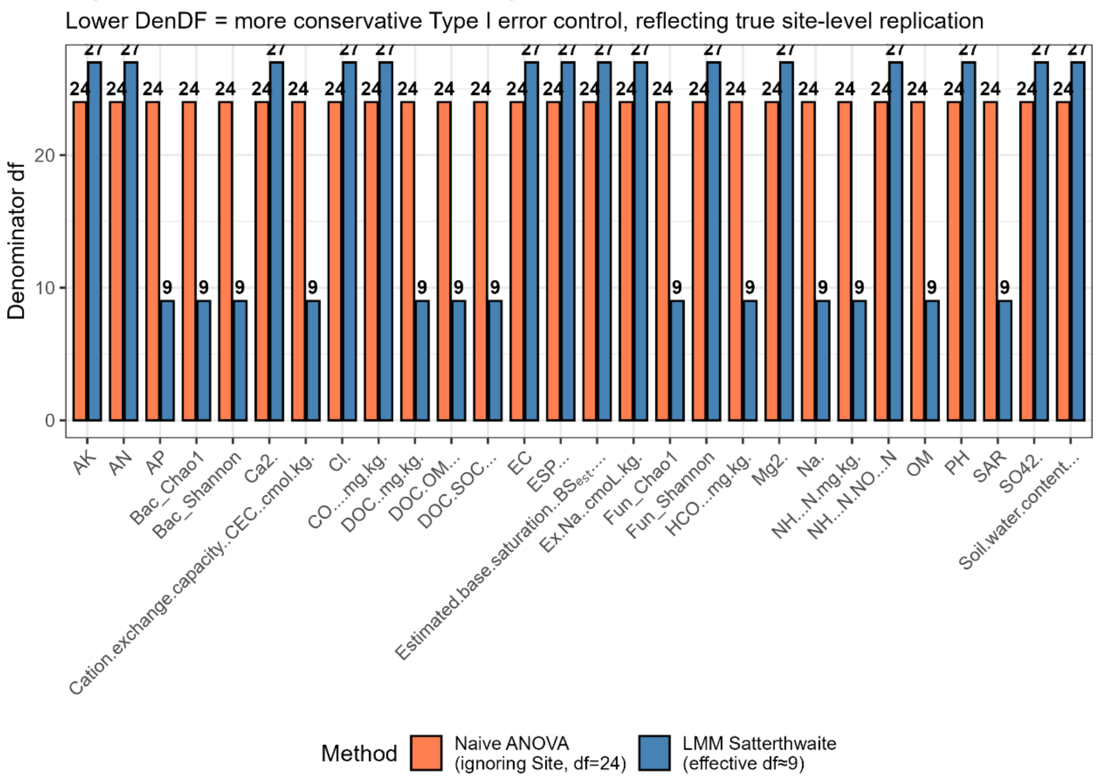

Figure S5. Conservative denominator degrees of freedom (DenDF) in LMM versus naive ANOVA. Orange bars: naive ANOVA ignoring Site (DenDF = 24, treating all 27 quadrats as independent). Blue bars: LMM with Site as a random effect and Satterthwaite DenDF. For variables with substantial site effects (e.g., Bac_Shannon, Bac_Chao1, AP), LMM yields conservative DenDF ≈ 9, reflecting true site-level replication (n = 9 sites). Naive ANOVA artificially inflates DenDF, thereby increasing Type I error risk.

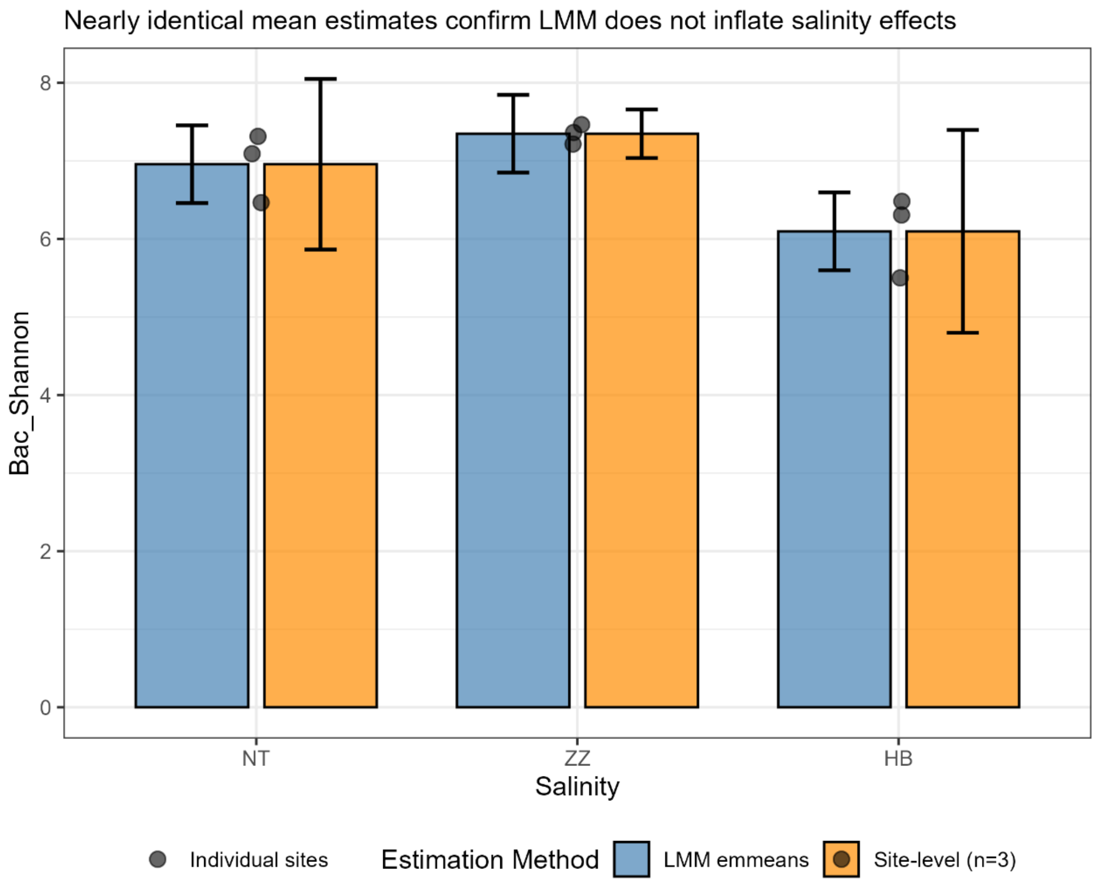

Figure S6. LMM emmeans versus site-level means for Bac_Shannon. Blue bars: estimated marginal means (emmeans) from LMM with 95% confidence intervals; orange bars: site-level means (n = 3 sites per salinity) with standard errors. Grey circles: individual site means. The near-identical estimates between LMM and site-level aggregation demonstrate that LMM does not inflate salinity effect sizes and that mean estimates are robust to the choice of analytical approach.

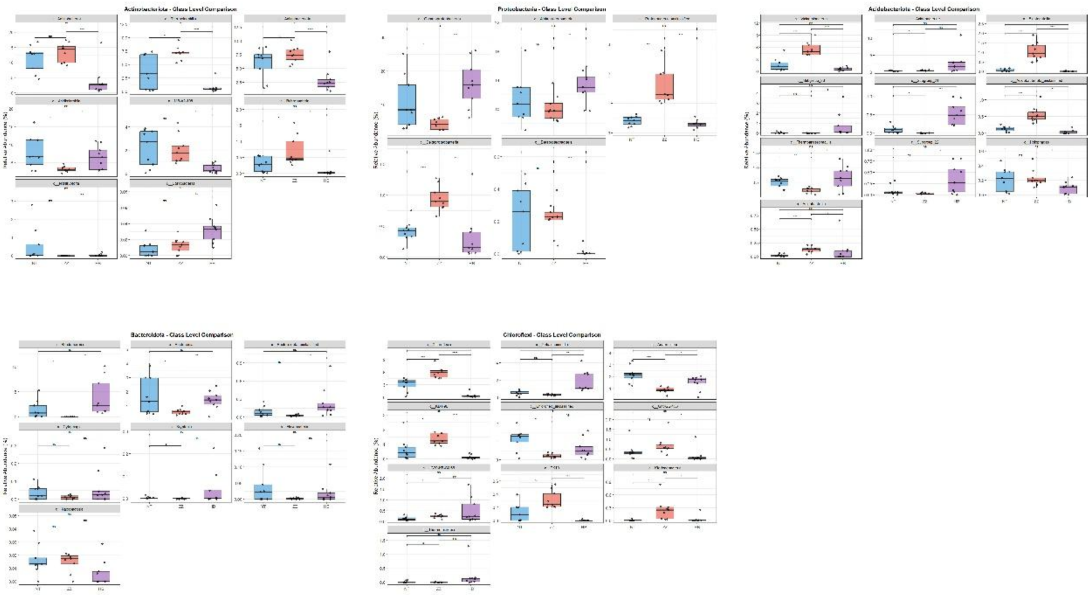

Figure S7. Significantly differentially abundant bacterial classes along the salinity gradient. Heatmap showing clr-transformed abundances; asterisks indicate significant differences among salinity groups (Kruskal–Wallis test with Benjamini–Hochberg FDR correction, adjusted $p < 0.05$). Complete statistical results are provided in Table S5.

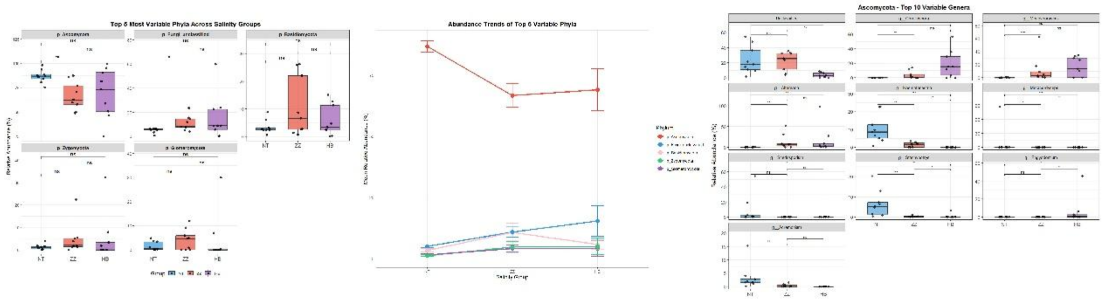

Figure S8. Statistical analysis of fungal community dynamics. Fungal community dynamics across salinity groups. Statistical significance for differential abundance was determined using Kruskal–Wallis tests on clr-transformed data with Benjamini–Hochberg FDR correction (ns: not significant at FDR-adjusted $p \geq 0.05$; * FDR-adjusted $p < 0.05$; ** FDR-adjusted $p < 0.01$; *** FDR-adjusted $p < 0.001$).

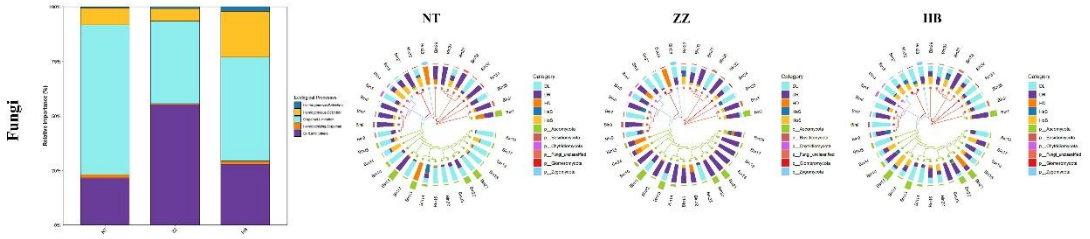

Figure S9. iCAMP analysis of community assembly processes at the phylogenetic-bin level (ds = 0.2). Stacked bars showing relative importance of five ecological processes for fungi: HeS (heterogeneous selection), HoS (homogeneous selection), DL (dispersal limitation), HD (homogenizing dispersal), and DR (drift). Because iCAMP operates at the bin level, taxonomic labels associated with specific processes indicate the dominant annotated lineage within each bin and do not imply that individual genera or species drive the corresponding process. Circular bar plots showing the relative contribution of each phylogenetic bin to the five assembly processes within each salinity group (NT, ZZ, HB). Bars are colored by process category (see legend). The inner dendrogram represents phylogenetic relationships among bins.

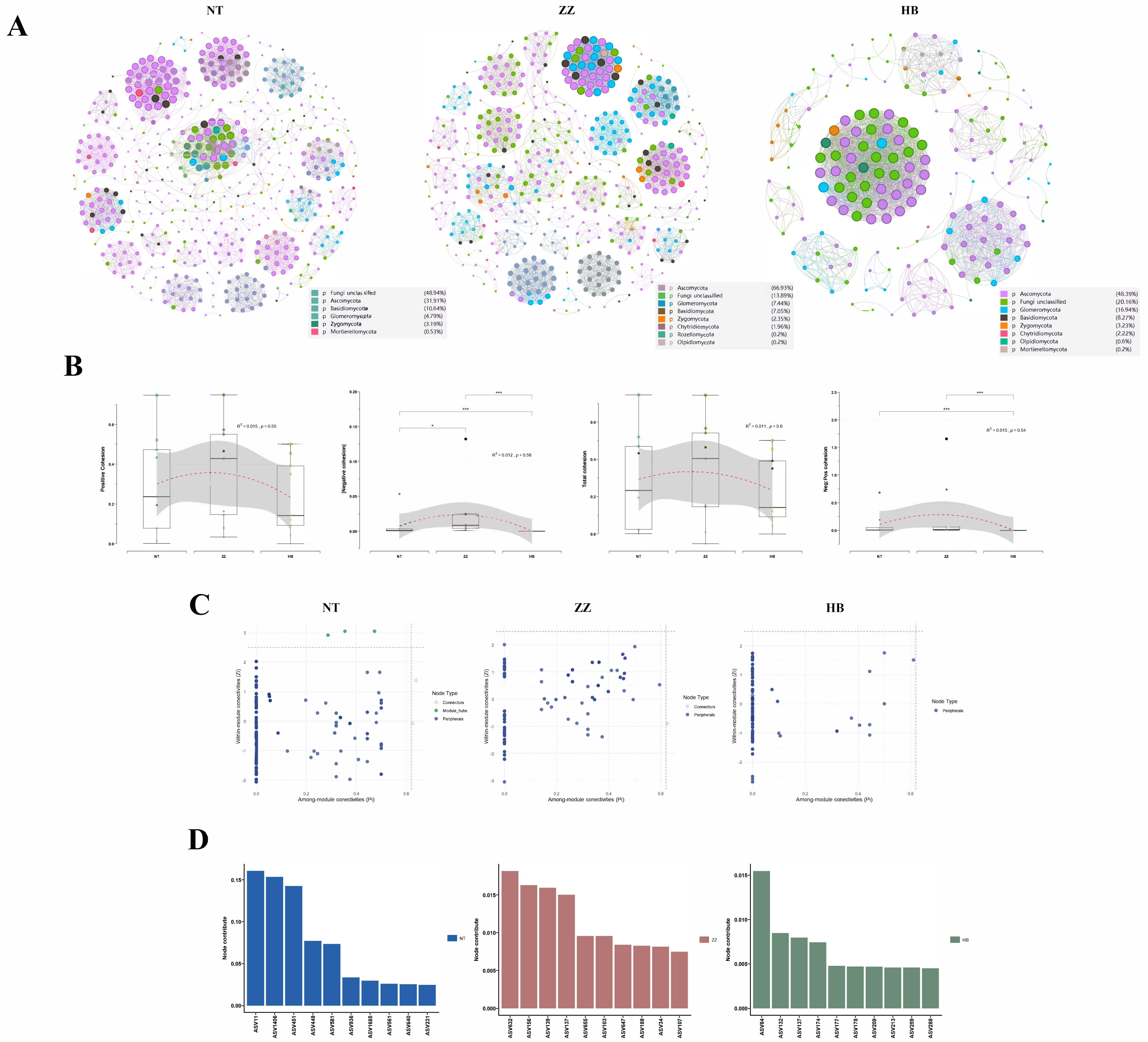

Figure S10. Topological evolution and destabilization of Fungi co-occurrence networks under saline-alkali stress.

(A) Visualization of microbial co-occurrence networks for NT, ZZ, and HB groups.

Nodes represent ASVs colored by phylum; edges represent significant strong correlations

(Spearman |r| > 0.8, P < 0.01). The fragmentation observed in the HB network illustrates the

"de-networking" effect induced by hypersalinity.

(B) Zi-Pi plots classifying the topological roles of nodes based on within-module connectivity (Zi) and among-module connectivity (Pi).

Nodes are categorized as peripherals, connectors, module hubs, or network hubs.

The scarcity of connectors and hubs in the HB group indicates a loss of core regulatory capacity.

(C) Bar charts showing the abundance contribution of the top 10 ASVs that structure the network.

(D) Analysis of community cohesion, displaying trends in

positive interaction, negative interaction, and total cohesion.

The peak in negative cohesion in the ZZ group suggests intensified competition,

while the overall decline in cohesion in the HB group reveals compromised community stability.
